# Ceruloplasmin correlates with immune infiltration and serves as a prognostic biomarker in breast cancer

**DOI:** 10.18632/aging.203427

**Published:** 2021-08-19

**Authors:** Fei Chen, Bihui Han, Yanxiu Meng, Yu Han, Bing Liu, Bo Zhang, Yanzhong Chang, Pengxiu Cao, Yumei Fan, Ke Tan

**Affiliations:** 1 Key Laboratory of Animal Physiology, Biochemistry and Molecular Biology of Hebei Province, College of Life Sciences, Hebei Normal University, Shijiazhuang 050024, Hebei, China

**Keywords:** ceruloplasmin, breast cancer, prognosis, immune infiltration, iron

## Abstract

Breast-invasive carcinoma (BRCA) is the most frequent and malignant tumor in females. Ceruloplasmin (CP) is a multifunctional molecule involved in iron metabolism, but its expression profile, prognostic potential and relationship with immune cell infiltration in BRCA are unknown. Ceruloplasmin mRNA and protein expression was significantly decreased in BRCA patients according to the Oncomine, UALCAN, GEPIA and TCGA databases. Ceruloplasmin expression was strongly correlated with various clinicopathological features of BRCA patients. BRCA patients with high ceruloplasmin expression exhibited shorter survival times than those with low ceruloplasmin expression based on the Kaplan-Meier plotter and PrognoScan databases. GO and KEGG analyses and GSEA revealed a strong correlation between ceruloplasmin and various immune-related pathways. Ceruloplasmin expression was significantly associated with the infiltration of immune cells into tumor sites by analyzing the TIMER and CIBERSORT. Additionally, ceruloplasmin was positively correlated with immune checkpoints in BRCA. These findings suggest that low ceruloplasmin expression correlates with a favorable prognosis and tumor immune cell infiltration in BRCA patients. Ceruloplasmin may serve as a therapeutic target and predict the efficacy of immunotherapy for BRCA.

## INTRODUCTION

Breast-invasive carcinoma (BRCA) is the most frequent malignant tumor in females worldwide [1]. Surgical resection is a curative treatment for BRCA patients in the early stage; however, many patients are still diagnosed at an advanced stage [2]. Other treatment strategies include chemotherapy, radiotherapy, endocrine agents and immunotherapy. Although these treatments have reduced the morbidity and mortality of BRCA patients, the prognosis of BRCA remains poor [2, 3]. The valuable prognostic biomarkers and therapeutic targets for BRCA remain to be elucidated [4].

Ceruloplasmin (CP), a multicopper oxidase, is a mammalian plasma ferroxidase [5]. Ceruloplasmin is mainly generated in the liver and then secreted into the circulation to reach other tissues and organs [6]. Approximately 40%-70% of the copper (Cu) found in plasma is carried by ceruloplasmin [7]. As a ferroxidase, ceruloplasmin could promote the conversion from Fe^2+^/Cu^1+^ to Fe^3+^/Cu^2+^, which are regarded as less toxic ion forms [8, 9]. Additionally, ceruloplasmin cooperates with ferroportin (FPN1), the iron efflux transporter, to export ferrous iron from cells [10, 11]. The human ceruloplasmin gene, comprising 1,046 amino acids with a total molecular weight of 132 kD, is located on chromosome 3q23-q24 [5–7]. It is well known that ceruloplasmin mainly exists in two forms in the human body, the secreted form and glycosylphosphatidylinositol (GPI)-linked form [12]. Secreted ceruloplasmin is primarily produced in hepatocytes and secreted into the plasma. By contrast, the GPI-anchored form of ceruloplasmin is ubiquitously expressed in various tissues and cells, including hepatocytes, macrophages, astrocytes, leptomeningeal cells and Sertoli cells [12–14]. To date, multiple activities and physiological roles of ceruloplasmin have been identified, including ferroxidase and antioxidant activity and the mediation of iron homeostasis, transportation of copper, and oxidation of organic amines [5]. Additionally, ceruloplasmin is also recognized as an acute-phase protein activated under different conditions, such as infection, inflammation, diabetes and trauma [15].

Recent evidence has indicated that ceruloplasmin is also correlated with tumor development and progression [16]. Elevated serum ceruloplasmin levels have been found in lung cancer, colon carcinoma, epithelial ovarian cancer and bile duct cancer [16–20]. Ceruloplasmin is associated with the invasiveness and prognosis of these cancer types through different molecular mechanisms and signaling pathways. By contrast, the expression of ceruloplasmin is significantly downregulated in adrenocortical carcinoma (ACC) and hepatocellular carcinoma and correlated with a poor prognosis in ACC patients [21, 22]. However, the expression pattern, prognostic value, DNA methylation and immune effects of ceruloplasmin in BRCA are still unknown.

In this context, we attempted to reveal the expression pattern, prognosis, methylation status and correlation of ceruloplasmin with immune cell infiltration to develop novel therapeutic strategies and prognostic biomarkers for BRCA. We conducted GO, KEGG and GSEA to identify the molecular mechanisms by which ceruloplasmin affects BRCA development and the biological signaling pathways in which ceruloplasmin may be involved. Additionally, we explored the correlations of ceruloplasmin expression with infiltration patterns for different tumor-infiltrating immune cells in BRCA.

## RESULTS

### Expression of ceruloplasmin in diverse human cancers

**Figure 1:**
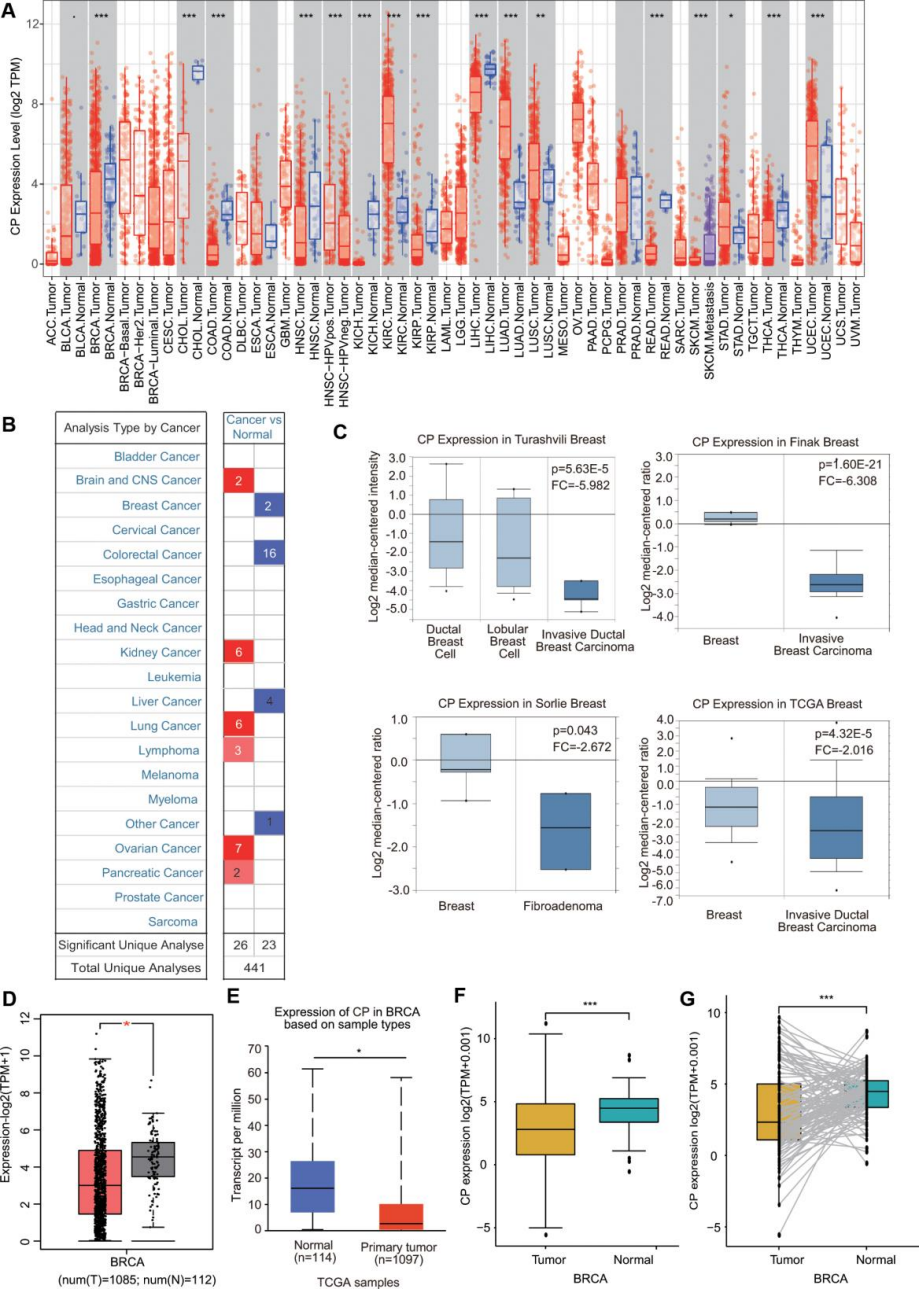
**Ceruloplasmin transcript levels in diverse cancer types.** (**A**) Differential ceruloplasmin mRNA expression between tumor tissues and normal tissues in the TIMER database. (**B**) Significant upregulation (red) and downregulation (blue) of ceruloplasmin mRNA in cancer tissues compared with normal tissues. (**C**) Box plots demonstrating ceruloplasmin expression in the Turashvili breast, Finak breast, Sorlie breast and TCGA breast datasets. (**D**, **E**) The mRNA levels of ceruloplasmin were lower in BRCA than in normal tissues in the GEPIA2 and UALCAN databases. (**F**) Ceruloplasmin expression levels in BRCA samples obtained from TCGA. (**G**) Ceruloplasmin expression in 112 matched BRCA and normal breast tissues in the TCGA database was examined. *<0.05, **<0.01, ***<0.001.

First, the mRNA levels of ceruloplasmin in common cancers and adjacent/normal tissues were investigated according to the TIMER online database. The expression of ceruloplasmin was obviously decreased in BRCA, cholangiocarcinoma (CHOL), colon adenocarcinoma (COAD), kidney chromophobe (KICH), kidney renal papillary cell carcinoma (KIRP), head and neck squamous cell carcinoma (HNSC), liver hepatocellular carcinoma (LIHC), rectum adenocarcinoma (READ) and thyroid carcinoma (THCA) tissues compared with normal tissues and was increased in cancer versus normal tissues in the kidney renal clear cell carcinoma (KIRC), lung adenocarcinoma (LUAD), lung squamous cell carcinoma (LUSC), stomach adenocarcinoma (STAD) and uterine corpus endometrial carcinoma (UCEC) tissues (Figure 1A). Oncomine analysis also suggested that the transcriptional levels of ceruloplasmin were obviously downregulated in multiple BRCA types, including invasive breast carcinoma, fibroadenoma, invasive ductal breast carcinoma, mixed lobular and ductal breast carcinoma, invasive lobular breast carcinoma and ductal breast carcinoma *in situ* with respect to normal breast tissues (Figure 1B, 1C and Supplementary Figure 1A). Consistent with the above results, ceruloplasmin was expressed at lower levels in BRCA tissues in the GEPIA and UALCAN databases (Figure 1D, 1E). Ceruloplasmin expression was also markedly decreased in BRCA using The Cancer Genome Atlas (TCGA) database (Figure 1F). Additionally, downregulated expression of ceruloplasmin was observed in 112 BRCA tissues compared with paired adjacent normal breast tissues (Figure 1G).

### Ceruloplasmin expression and clinicopathologic parameters in BRCA patients

**Figure 2:**
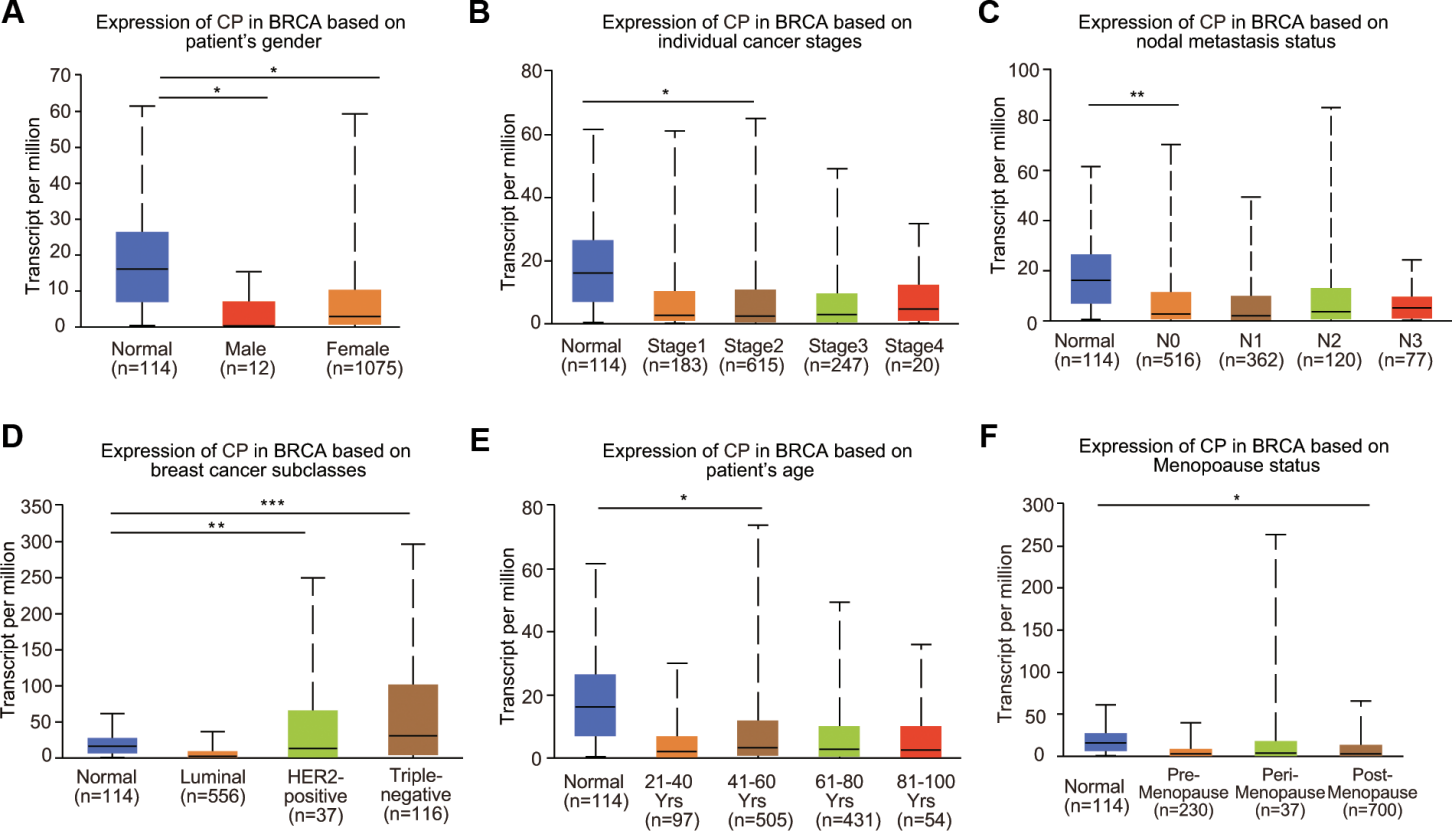
**Relationship between ceruloplasmin mRNA expression and clinicopathological features of BRCA patients.** Ceruloplasmin expression was estimated in BRCA patients according to (**A**) sex, (**B**) different cancer stages, (**C**) different nodal metastasis statuses, (**D**) major BRCA subclasses, (**E**) age and (**F**) menopausal status. *<0.05, **<0.01, ***<0.001.

To further clarify the expression profiles of ceruloplasmin in BRCA, we investigated the relationship between ceruloplasmin and various clinicopathological parameters. Mining of the UALCAN database suggested that ceruloplasmin expression was decreased in both male and female BRCA patients (Figure 2A). We further analyzed ceruloplasmin expression based on individual cancer stages and found that ceruloplasmin expression was closely correlated with stage 2 BRCA (Figure 2B). According to the nodal metastasis status, ceruloplasmin expression was also dramatically reduced in the N0 stage but not in the N1, N2 and N3 stages in BRCA patients (Figure 2C). When divided into subclasses, lower expression of ceruloplasmin was observed in HER2-positive BRCA patients than in other patients (Figure 2D). Ceruloplasmin was decreased in the 41- to 60-year-old age group compared with the normal group (Figure 2E). Ceruloplasmin expression was significantly decreased in postmenopausal patients, but not in premenopausal or perimenopausal patients. Additionally, no other differences were found based on the premenopausal, perimenopausal or postmenopausal status (Figure 2F). Ceruloplasmin expression was also significantly downregulated in African-Americans (Supplementary Figure 1B).

**Figure 3:**
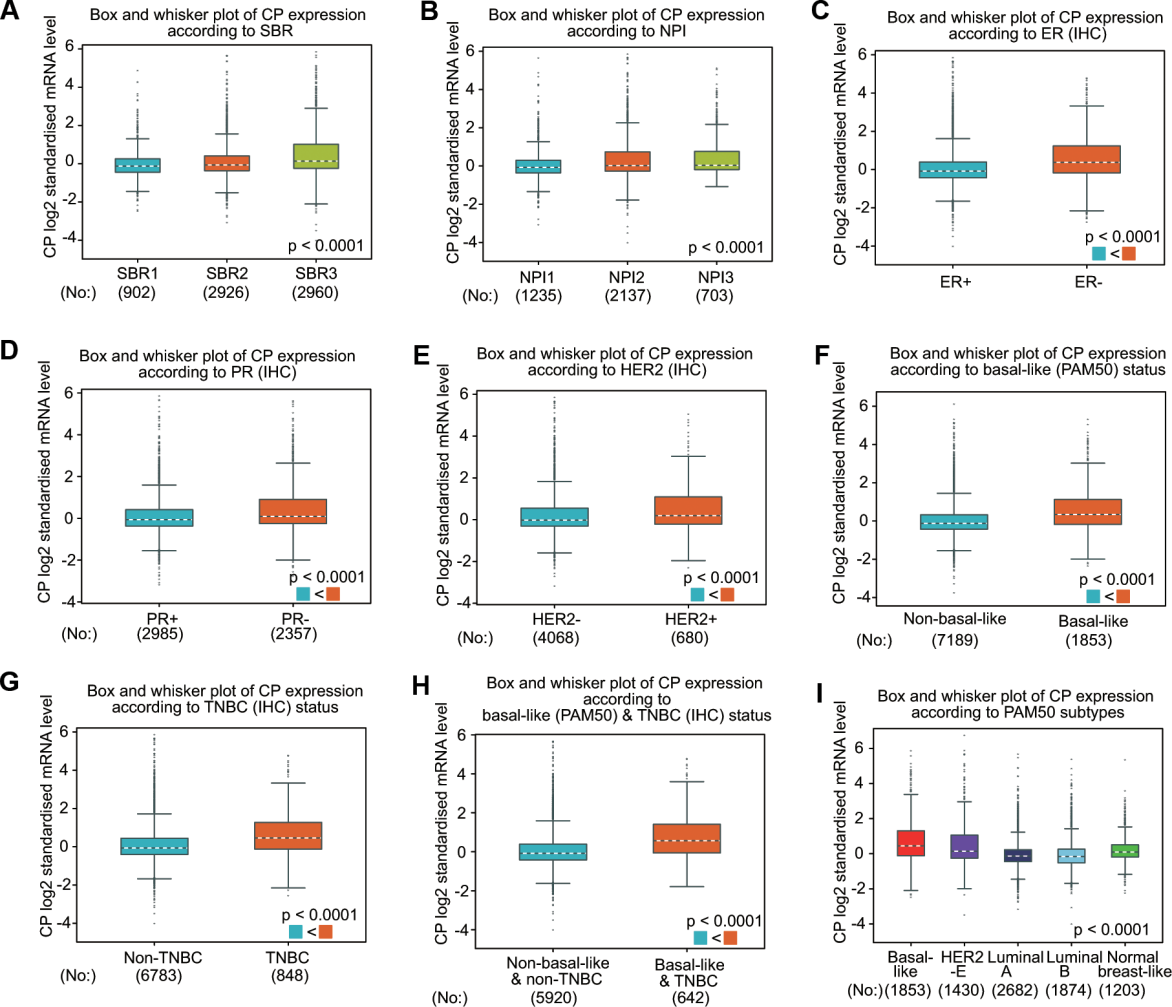
**Ceruloplasmin expression in BRCA patients based on various pathological characteristics was assessed using bc-GenExMiner v4.5.** Box plots are shown for the SBR grade (**A**), NPI index (**B**), status of ER (**C**), PR (**D**) and HER-2 (**E**), basal-like (**F**), TNBC (**G**), basal-like and TNBC status (**H**) and PAM50 subtypes (**I**).

The associations between ceruloplasmin expression and the clinical parameters of BRCA patients were further confirmed through the bc-GenExMiner database (Figure 3). BRCA patients with more advanced Nottingham prognostic index (NPI) values and Scarff-Bloom-Richardson (SBR) grade expressed higher levels of ceruloplasmin mRNA (Figure 3A, 3B). The ceruloplasmin expression level in estrogen receptor (ER)-positive or progesterone receptor (PR)-positive BRCA patients was much lower than that in ER-negative or PR-negative BRCA patients (Figure 3C, 3D). By contrast, the expression of ceruloplasmin was decreased in HER2-negative BRCA patients compared with that in HER2-positive BRCA patients (Figure 3E). Ceruloplasmin expression was also dramatically downregulated in non-triple-negative breast cancer (TNBC) and non-basal-like BRCA patients compared with that in basal-like and TNBC BRCA patients (Figure 3F–3H). BRCA patients with the luminal A subtype and luminal B subtype exhibited lower ceruloplasmin expression (Figure 3I). Taken together, these findings reveal that ceruloplasmin expression is widely associated with diverse clinicopathological characteristics in BRCA.

### Protein level of ceruloplasmin in BRCA

**Figure 4:**
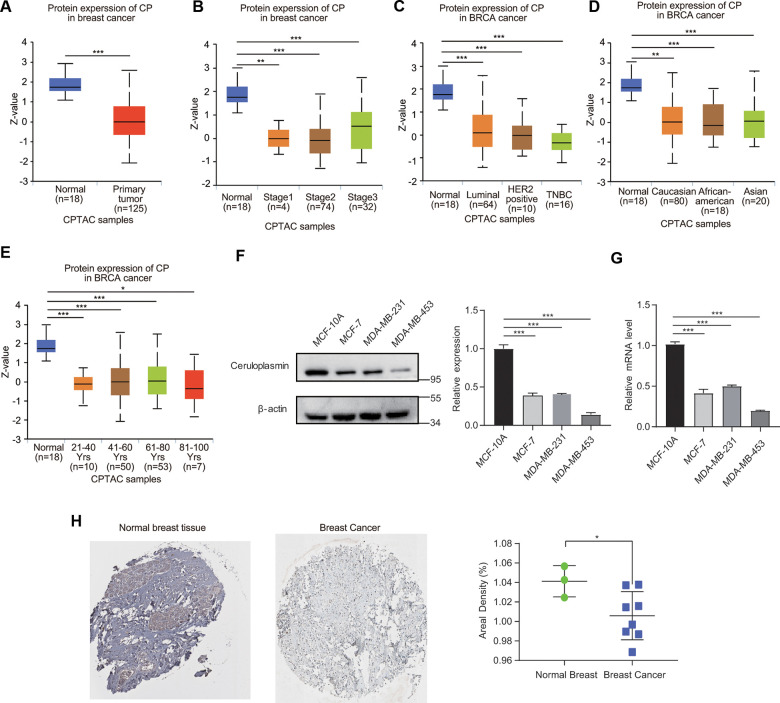
**Ceruloplasmin protein levels in BRCA.** (**A**) Ceruloplasmin protein levels in BRCA tissues and normal breast tissues were investigated based on the CPTAC database. (**B**–**E**) Relationship between ceruloplasmin protein expression and clinicopathological parameters of BRCA patients, including individual cancer stage, major subclass, patient race and age. (**F**) The protein levels of ceruloplasmin in MCF-10A, MDA-MB-231 and MCF-7 cells were investigated by western blotting. (**G**) The mRNA levels of ceruloplasmin in three different cell lines were examined by real-time PCR. (**H**) Representative IHC images of ceruloplasmin in normal breast tissues (upper) and BRCA (lower) were shown. The staining was quantified (normal breast tissue, n=3; BRCA, n=8). One-way ANOVA was used. *<0.05, **<0.01, ***<0.001.

We then evaluated the protein expression level of ceruloplasmin in BRCA. The ceruloplasmin protein levels were obviously decreased in BRCA samples compared with normal samples according to the UALCAN database (Figure 4A). Consistent with the change observed in the mRNA expression pattern, the ceruloplasmin protein level was also remarkably downregulated in BRCA patients across different disease stages (stages 1, 2 and 3), races (Caucasian, African-American and Asian), ages (21-40, 41-60, 61-80 and 81-100 years) and disease subtypes (luminal, HER2-positive and TNBC) (Figure 4B–4E).

### Ceruloplasmin expression in BRCA cell lines and clinical specimens

We first estimated the protein level of ceruloplasmin in nonmalignant breast epithelial cells (MCF-10A) and three BRCA cell lines (MCF-7, MDA-MB-453 and MDA-MB-231). We found that ceruloplasmin protein expression was much lower in MCF-7, MDA-MB-231 and MDA-MB-453 cells than in MCF-10A cells (Figure 4F). Consistent with the protein level, the mRNA level of ceruloplasmin was also lower in BRCA cells compared with normal breast cancer cells (Figure 4G). Additionally, the reduced protein expression of ceruloplasmin was confirmed by IHC staining (Figure 4H). These results confirm the decreased expression of ceruloplasmin in BRCA.

### Prognostic value of ceruloplasmin in BRCA patients

**Figure 5:**
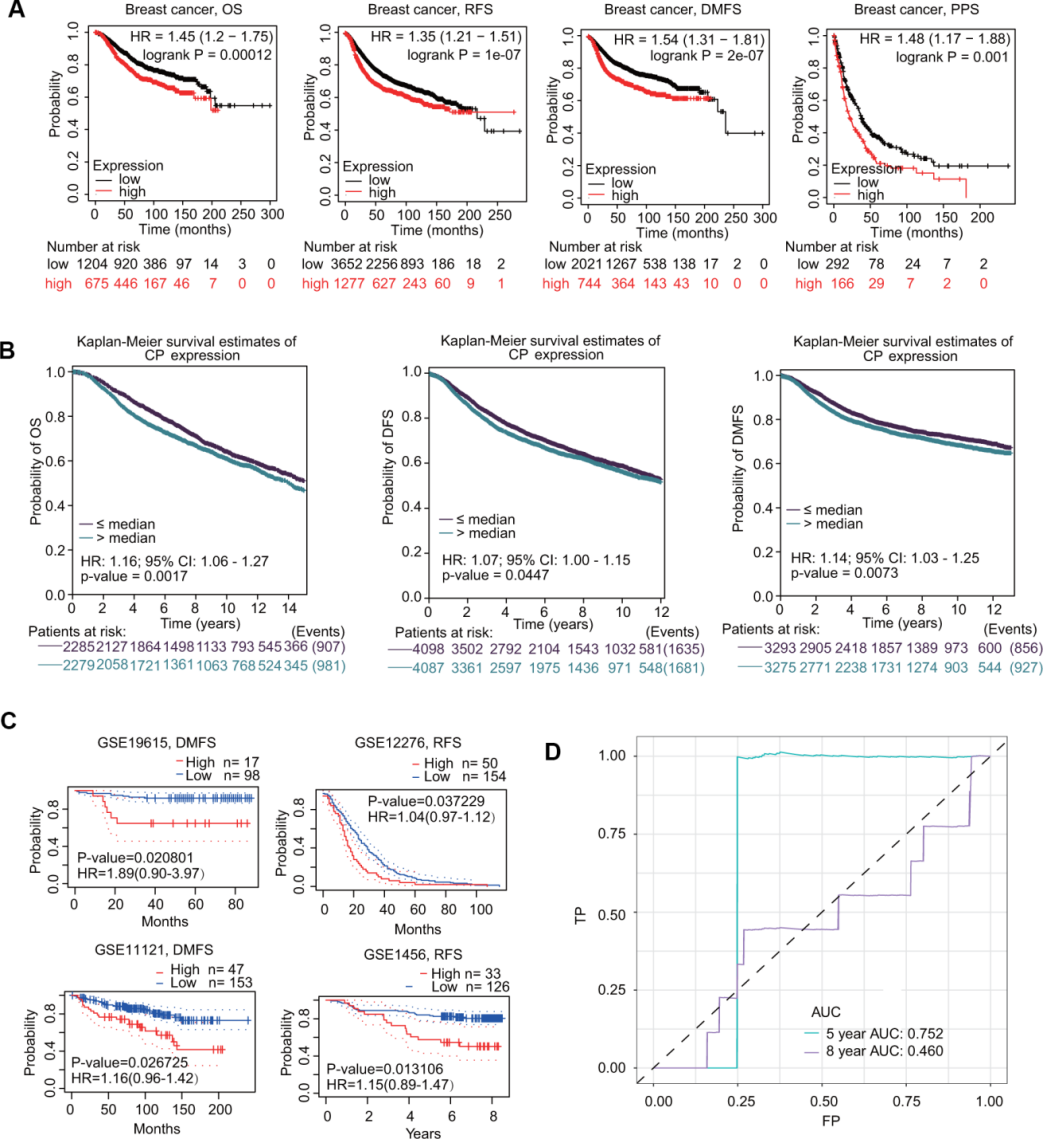
**Prognostic values of ceruloplasmin in BRCA patients.** (**A**) Survival curves from Kaplan–Meier Plotter indicated that higher ceruloplasmin expression was related to worse OS, RFS, DMFS and PPS in patients with BRCA. (**B**) Survival curves from bc-GenExMiner v4.5 showed the prognostic value of ceruloplasmin in BRCA patients based on OS, DFS and DMFS. (**C**) The DMFS and RFS in BRCA cohorts using the PrognoScan database. (**D**) ROC curve of ceruloplasmin mRNA expression in BRCA.

The prognostic value of ceruloplasmin in BRCA was first assessed using the Kaplan-Meier plotter database. As shown in Figure 5A, higher expression of ceruloplasmin was correlated with worse overall survival (OS) (HR=1.45; p=0.00012), relapse-free survival (RFS) (HR=1.35; p=1e-07), distant metastasis-free survival (DMFS) (HR=1.54; p=2e-07) and postprogression survival (PPS) (HR=1.48; p=0.001) in BRCA patients. Subsequently, the prognostic potential of ceruloplasmin in BRCA was investigated using the bc-GenExMiner online service. Upregulated expression of ceruloplasmin was closely associated with reduced OS, disease-free survival (DFS) and DMFS time in BRCA (Figure 5B). Consistent results were validated in the PrognoScan database. Assessment of four different cohorts (GSE19615, GSE12276, GSE11121 and GSE1456) including different types of BRCA revealed that upregulated ceruloplasmin expression was significantly linked with poor DMFS and RFS in BRCA patients (Figure 5C). To further assess the diagnostic potential of ceruloplasmin expression in BRCA, ROC (receiver operating characteristic) curve analysis was performed. The value of AUC (area under the curve) was 0.752 for 8-year survival (Figure 5D), indicating that ceruloplasmin exhibits considerable diagnostic value in distinguishing between BRCA and noncancerous breast tissues.

### Methylation level and genetic alteration of ceruloplasmin in BRCA

**Figure 6:**
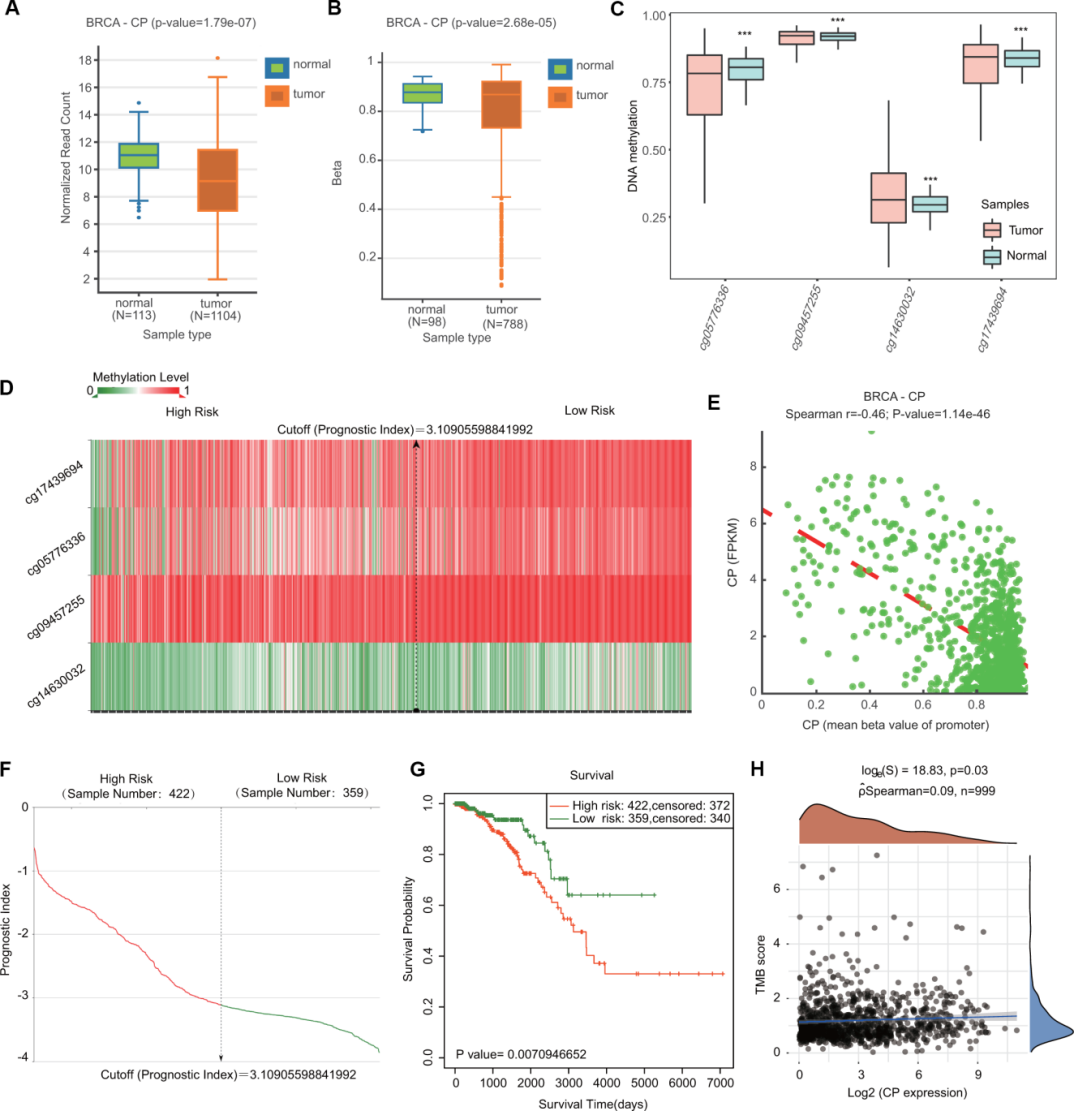
**Comparison of the DNA methylation status of ceruloplasmin in BRCA and adjacent breast tissues.** (**A**, **B**) Expression and methylation levels of CP in BRCA through the DNMIVD database. (**C**) Methylation levels of CP in BRCA using the SurvivalMeth database. (**D**) Heat map of DNA methylation of CP in BRCA. (**E**) Correlation between CP expression and CpG site methylation levels. (**F**) Distribution of the prognostic index. (**G**) Prognostic potential of DNA methylation of CP in BRCA from the SurvivalMeth database. (**H**) Correlation between CP expression and TMB in BRCA.

Because DNA methylation is important for tumor initiation and progression, we next examined the DNA methylation of ceruloplasmin through the DNMIVD and SurvivalMeth databases. Compared with normal samples, BRCA samples exhibited lower levels of ceruloplasmin and DNA methylation in both the gene body and promoter regions (Figure 6A, 6B). We then investigated the methylation levels of four CpG sites (cg05776336, cg09457255, cg14630032 and cg17439694) in the DNA of the ceruloplasmin gene. Three of the CpG sites were significantly less methylated in BRCA tissues than in adjacent breast tissues (Figure 6C). The heat map of the DNA methylation results for ceruloplasmin in BRCA is shown in Figure 6D. The methylation levels of these CpG sites were negatively correlated with ceruloplasmin mRNA expression (Figure 6E). The effect of ceruloplasmin DNA methylation on the survival of BRCA patients was explored via the SurvivalMeth and MethSurv databases. Higher methylation levels at the CpG sites of the ceruloplasmin gene were significantly correlated with a poor prognosis in BRCA (Figure 6G).

The relationships between the expression of ceruloplasmin and TMB/MSI were also evaluated. Ceruloplasmin expression was markedly associated with TMB, not MSI, in BRCA (Figure 6H and Supplementary Figure 2A). The alteration frequency of ceruloplasmin was assessed through the cBioPortal database. Ceruloplasmin had an alternation frequency of 2.4%, with amplification being the most common variation in ceruloplasmin (Supplementary Figure 2B, 2C). Survival curve analysis demonstrated that BRCA patients with genetic variations in ceruloplasmin exhibited poor OS and RFS, but the difference was not statistically significant (Supplementary Figure 2D).

### Neighboring gene network of ceruloplasmin in BRCA

**Figure 7:**
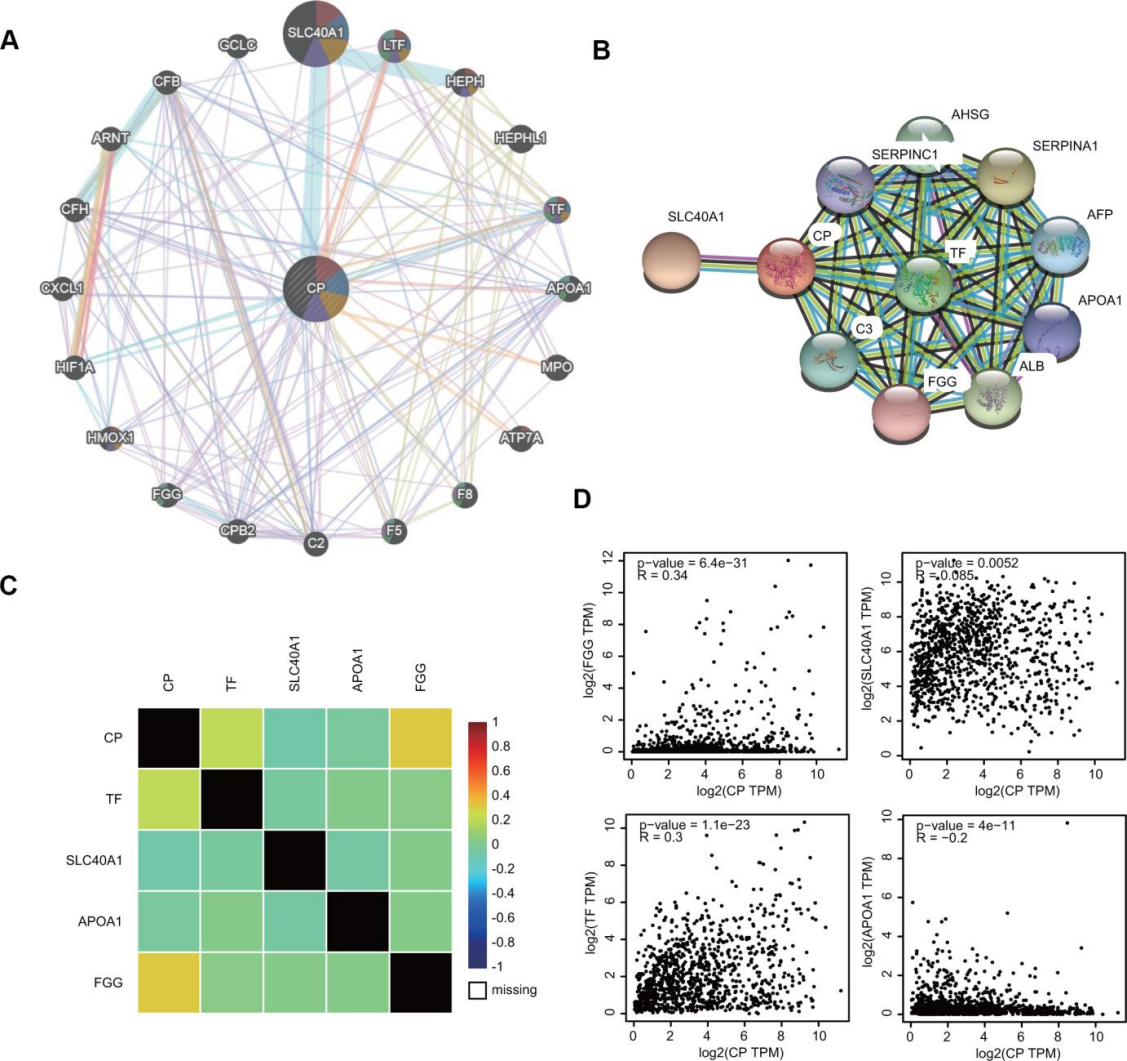
**Interaction between ceruloplasmin and related genes and proteins.** (**A**, **B**) The gene-gene interactive network and PPI of ceruloplasmin were generated using the GeneMANIA and STRING databases, respectively. (**C**) The correlations between ceruloplasmin expression and TF, SLC40A1, APOA1 and FGG expression were obtained from the bc-GenExMiner v4.5 database. (**D**) The correlations between ceruloplasmin expression and TF, SLC40A1, APOA1 and FGG expression were obtained from the GEPIA2 database.

To investigate the ceruloplasmin-related gene interaction network, we searched for altered neighboring genes using the GeneMANIA online database. Twenty genes, including several iron metabolic genes (SLC40A1, HEPH, TF and HMOX1), were closely correlated with ceruloplasmin (Figure 7A). We further constructed a protein-protein interaction (PPI) network to identify ceruloplasmin-interacting proteins through the STRING database. Among the 11 nodes in the PPI network, four genes (TF, SLC40A1, APOA1 and FGG) were identified from the databases (Figure 7B). The relationship between ceruloplasmin and these four genes was evaluated using the bc-GenExMiner database (Figure 7C). As respected, the expression of these four genes was notably associated with the expression of ceruloplasmin in the GEPIA2 database (Figure 7D).

### Functional and signaling pathway enrichment analyses for ceruloplasmin

**Figure 8:**
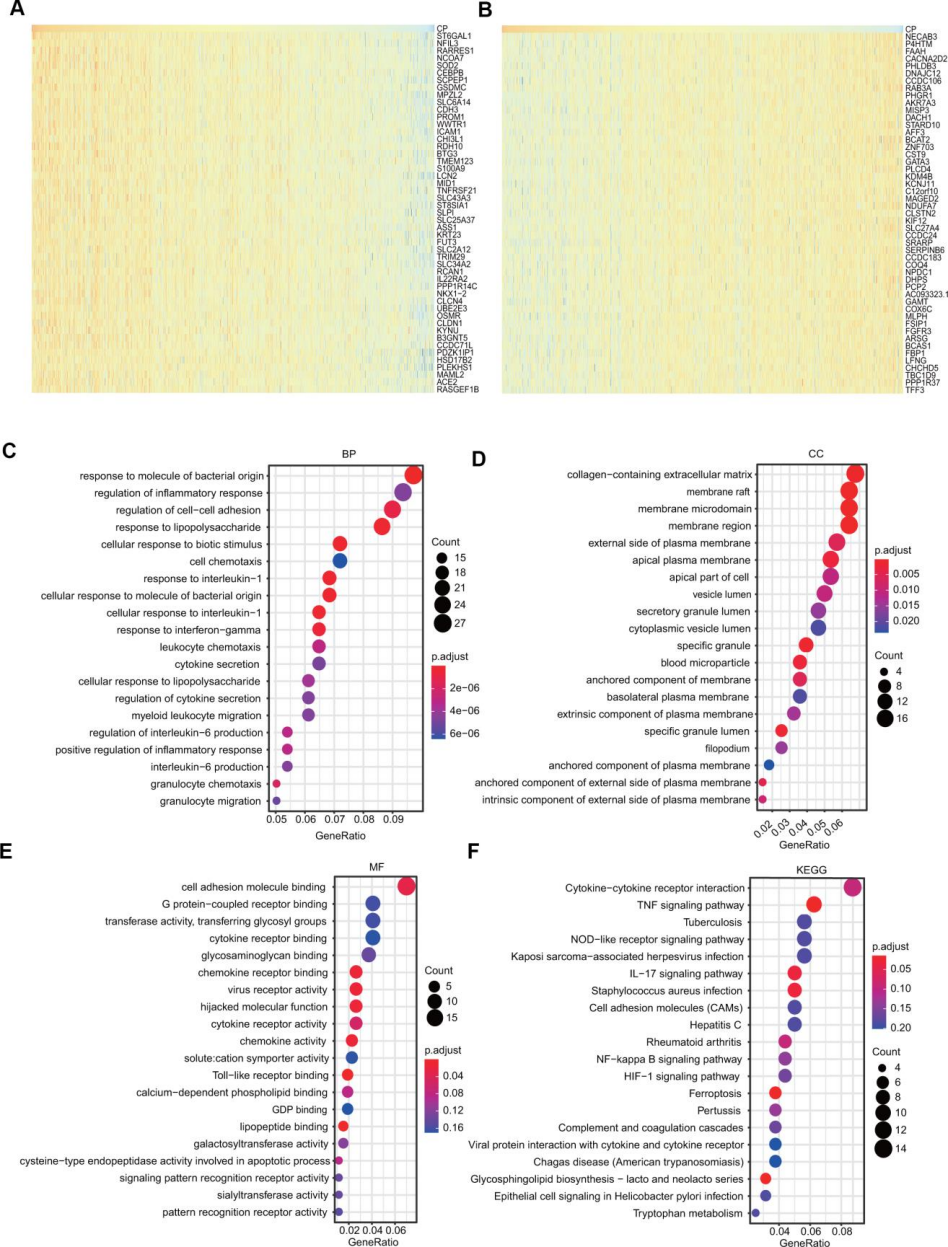
**GO and KEGG analyses of ceruloplasmin in the TCGA-BRCA cohort.** (**A**, **B**) Heat maps demonstrating the top 50 genes positively or negatively linked with ceruloplasmin in TCGA-BRCA. (**C**–**E**) Twenty significantly enriched GO annotations (BP, MF and CC) of ceruloplasmin in the TCGA-BRCA cohort. (**F**) Significantly enriched 20 KEGG pathways of ceruloplasmin in the TCGA-BRCA cohort.

The genes positively coexpressed with ceruloplasmin were utilized to analyze functional and signaling pathway enrichment. The heatmaps showed the top 50 genes that were positively or negatively correlated with ceruloplasmin in BRCA (Figure 8A, 8B). Both GO and KEGG analyses are powerful bioinformatics tools to assess the molecular function of ceruloplasmin. Bubble plots were generated to show the top 20 enriched biological processes (BPs), molecular functions (MFs) and cell components (CCs) terms from the GO enrichment analysis (Figure 8C–8E). Notably, among the BP terms, many immune response-related pathways were closely associated with ceruloplasmin, such as the response to molecules of bacterial origin, regulation of the inflammatory response, response to lipopolysaccharide, response to interleukin-1, response to interferon-gamma, cytokine secretion and the regulation of interleukin-6 production (Figure 8C). Similarly, regarding the MF terms, ceruloplasmin was significantly correlated with inflammatory response pathways, including cytokine receptor binding, chemokine receptor binding, cytokine receptor activity and Toll-like receptor binding (Figure 8E). KEGG pathway analysis further confirmed that ceruloplasmin was involved in the inflammatory response, as multiple immune-related pathways (TNF signaling pathway, cytokine-cytokine receptor interaction, IL-17 signaling pathway, and NOD-like receptor signaling pathway) were found to be associated with CP (Figure 8F).

### Ceruloplasmin-associated pathways identified by GSEA

**Figure 9:**
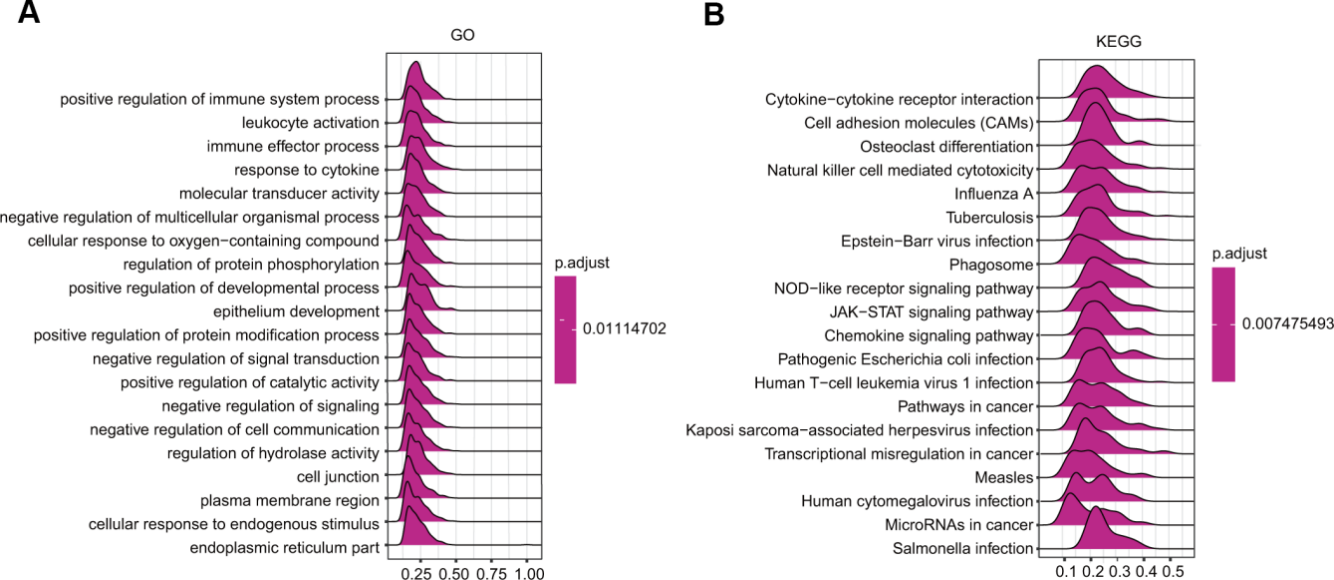
GSEA results revealing the ceruloplasmin-associated signaling pathways based on KEGG (**A**) and Reactome analyses (**B**) in BRCA.

GSEA was further applied to predict the ceruloplasmin-related signaling pathways that were differentially activated in BRCA. Regarding the GO terms, the top 4 pathways affected by ceruloplasmin (positive regulation of immune system processes, leukocyte activation, immune effector processes and response to cytokines) were all correlated with the immune response (Figure 9A and Supplementary Table 1). Among the KEGG terms, the outcome of GSEA also suggested that different immune functional pathways, including natural killer cell-mediated cytotoxicity, cytokine-cytokine receptor interaction, and various bacterial or viral infections, were enriched in BRCA (Figure 9B and Supplementary Table 1). Taken together, these results strongly imply a close relationship among ceruloplasmin, the inflammatory response and the tumor microenvironment (TME).

### Immune cell infiltration of ceruloplasmin in BRCA patients

**Figure 10:**
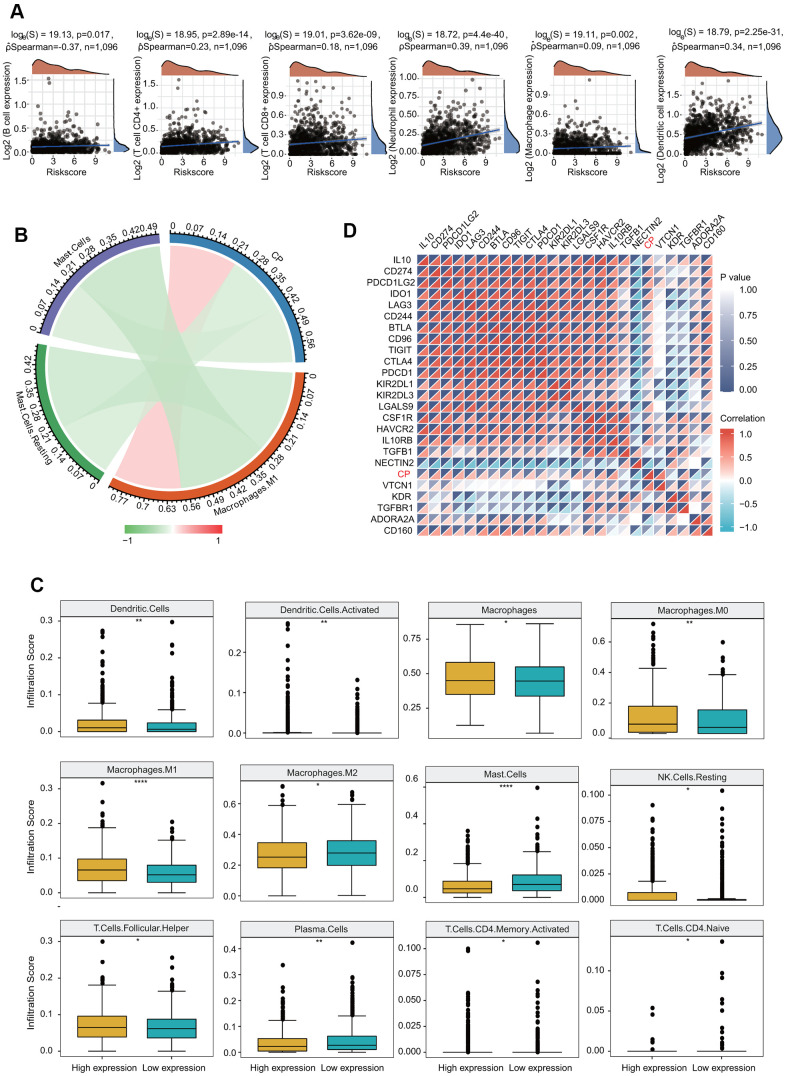
**Significant correlation of ceruloplasmin with the immune filtration level in BRCA.** (**A**) Correlation between the expression of ceruloplasmin and the level of immune infiltration through the TIMER database. (**B**) Analysis of the relationship between ceruloplasmin and immune infiltration level in the TCGA-BRCA dataset using CIBERSORT. (**C**) Twelve types of tumor-infiltrating immune cells are plotted based on ceruloplasmin expression using CIBERSORT. (**D**) Correlations between ceruloplasmin expression and immune checkpoint genes in BRCA using the R software package ggstatsplot.

We then evaluated the correlations of ceruloplasmin with the infiltration of immune cells in BRCA. Ceruloplasmin was notably associated with tumor purity in BRCA. Additionally, ceruloplasmin was markedly and positively linked with the infiltration levels of the six major types of immune cells examined in BRCA in the TIMER database (Figure 10A). Based on the different subtypes of BRCA, we observed that ceruloplasmin expression was significantly associated with the infiltration abundances of CD4+ T cells, CD8+ T cells, neutrophils, macrophages, and dendritic cells in luminal A breast cancer (Supplementary Figure 3A). Ceruloplasmin expression was remarkably associated with the infiltration abundances of CD8+ T cells, neutrophils, and dendritic cells in luminal B breast cancer (Supplementary Figure 3B). Ceruloplasmin expression was also associated with the infiltration levels of CD4+ T cells, neutrophils, and dendritic cells in basal-like breast cancer (Supplementary Figure 3C). Ceruloplasmin expression was only associated with the infiltration abundances of CD8+ T cells in normal-like breast cancer (Supplementary Figure 3D). Finally, there is no significant relationship was found between ceruloplasmin expression and the the infiltration levels of these immune cells in HER2-enriched breast cancer (Supplementary Figure 3E). Next, the correlation between ceruloplasmin and the infiltration level of different immune cells was assessed using CIBERSORT (Figure 10B). Ceruloplasmin was positively and dramatically linked with the infiltrating abundances of native B cells, dendritic cells, activated dendritic cells, M0 and M1 macrophages, CD4 memory T cells and follicular helper T (TfH) cells (Figure 10B and Supplementary Figure 4A) and was negatively linked with the infiltration abundances of monocytes, mast cells, M0 macrophages and plasma cells in BRCA (Figure 10B and Supplementary Figure 4B). Decreased ceruloplasmin expression was dramatically associated with low infiltrating levels of activated dendritic cells, dendritic cells, macrophages, M0 and M1 macrophages, resting NK cells and TfH cells but with high infiltration levels of M2 macrophages, resting mast cells, mast cells, activated memory CD4 T cells, naïve CD4 T cells, and plasma cells (Figure 10C).

### Ceruloplasmin expression is correlated with multiple immune signatures

Based on diverse immune cell gene marker sets, we studied the correlation of ceruloplasmin and infiltrating immune cells in BRCA. As shown in Tables 1, 2, we observed significant correlations between the expression of ceruloplasmin and most gene marker sets of different immune cells in both the TIMER and GEPIA2 databases. Additionally, ceruloplasmin was also associated with the infiltrating levels of T cell subtypes using the TIMER database. The expression of ceruloplasmin was dramatically associated with 48 of 54 and 46 of 54 markers of T cell in BRCA before and after adjustments for tumor purity, respectively (Table 3). More importantly, ceruloplasmin expression was positively and dramatically linked with the expression of most immune checkpoint genes, such as PD-L1, PD-1 and CTLA-4 (Figure 10D). The relationship between ceruloplasmin and these three well-known immune checkpoints was further evaluated according to the GEPIA2 and TIMER databases (Supplementary Figure 4C).

**Table 1:** Correlation analyses between ceruloplasmin and different genes of immune cells in TIMER.

**Description**	**Gene markers**	**BC**			
		**None**		**Purity**	
		**Cor**	**P**	**Cor**	**P**
**CD8+ T cell**	CD8A	0.164	***	0.061	0.056
	CD8B	0.231	***	0.144	***
**T cell (general)**	CD3D	0.208	***	0.096	**
	CD3E	0.201	***	0.084	**
	CD2	0.215	***	0.112	***
**B cell**	CD19	0.199	***	0.097	**
	CD79A	0.171	***	0.053	0.0977
**Monocyte**	CD86	0.25	***	0.193	***
	CSF1R	0.209	***	0.115	***
**TAM**	CCL2	0.312	***	0.253	***
	CD68	0.225	***	0.176	***
	IL10	0.212	***	0.161	***
**M1**	IRF5	0.089	**	0.048	0.127
	NOS2	0.021	0.479	0.008	0.81
	PTGS2	0.32	***	0.249	***
**M2**	CD163	0.246	***	0.199	***
	VSIG4	0.196	***	0.14	***
	MS4A4A	0.202	***	0.14	***
**Neutrophil**	CEACAM8	0.08	**	0.071	*
	ITGAM	0.217	***	0.153	***
	CCR7	0.2	***	0.093	**
**Natural**	KIR2DL1	0.116	***	0.074	*
**killer cell**	KIR2DL3	0.163	***	0.117	***
	KIR2DL4	0.227	***	0.178	***
	KIR3DL1	0.187	***	0.118	***
	KIR3DL2	0.197	***	0.129	***
	KIR3DL3	0.077	*	0.041	0.194
	KIR2DS4	0.137	***	0.087	**
**Dendritic cell**	HLA-DPB1	0.166	***	0.037	0.245
	HLA-DQB1	0.187	***	0.096	**
	HLA-DRA	0.25	***	0.16	***
	HLA-DPA1	0.201	***	0.105	***
	CD1C	0.138	***	-0.012	0.717
	NRP1	0.177	***	0.103	**
	ITGAX	0.214	***	0.137	***

**Table 2:** Correlation analyses between ceruloplasmin and gene markers of immune cells in GEPIA.

**Description**	**Gene markers**	**BC**	
		**Purity**	
		**R**	**P**
**B cell**	CD19	0.19	***
	CD79A	0.15	***
**T cell (general)**	CD3D	0.19	***
	CD3E	0.19	***
	CD2	0.2	***
**CD8+T cell**	CD8A	0.15	***
	CD8B	0.22	***
**Monocyte**	CD86	0.24	***
	CSF1R	0.2	***
	CD68	0.22	***
**TAM**	CCL2	0.31	***
	IL10	0.18	***
**M1**	IRF5	0.087	**
	NOS2	0.025	0.42
	PTGS2	0.31	***
**M2**	CD163	0.25	***
	VSIG4	0.19	***
	MS4A4A	0.19	***
**Neutrophil**	CEACAM8	0.056	0.065
	ITGAM	0.22	***
	CCR7	0.19	***
**Dendritic cell**	HLA-DPA1	0.2	***
	HLA-DPB1	0.17	***
	HLA-DQB1	0.15	***
	HLA-DRA	0.25	***
	CD1C	0.13	***
	NRP1	0.17	***
	ITGAX	0.21	***
**Natural killer cell**	KIR2DL1	0.16	***
	KIR2DL3	0.15	***
	KIR2DL4	0.22	***
	KIR2DS4	0.13	***
	KIR3DL1	0.15	***
	KIR3DL2	0.22	***

**Table 3:** Correlation analyses between ceruloplasmin and gene markers of various T cells in TIMER.

**Description**	**Gene markers**	**BC**			
		**None**		**Purity**	
		**Cor**	**P**	**Cor**	**P**
**Th1**	TBX21	0.208	***	0.103	**
	STAT4 STAT1	0.255	***	0.164	***
		0.19	***	0.166	***
	IFNG	0.209	***	0.15	***
	TNF	0.262	***	0.227	***
**Th2**	GATA3	0.435	***	-0.402	***
	STAT6	-0.056	0.065	-0.106	***
	STAT5A	0.121	***	0.051	0.11
	IL13	0.143	***	0.107	***
**Tfh**	BCL6	0.234	***	0.203	***
	IL21	0.127	***	0.073	*
**Th17**	STAT3	0.357	***	0.357	***
	IL17A	0.068	**	0.058	0.065
**Treg**	FOXP3	0.195	***	0.112	***
	CCR8	0.182	***	0.139	***
	STAT5B	-0.01	0.734	-0.029	0.367
**Effector T-cell**	TGFB1	-0.005	0.866	-0.12	***
	CX3CR1	0.008	0.789	-0.063	*
	FGFBP2	0.222	***	0.129	***
**Naïve T-cell**	FCGR3A	0.185	***	0.158	***
	CCR7	0.2	***	0.093	**
	SELL	0.192	***	0.086	**
	TCF7	0.305	***	0.225	***
**Effective memory T-cell**	LEF1	0.022	0.457	-0.04	0.204
	PDCD1	0.213	***	0.117	***
	DUSP4	-0.087	**	-0.131	***
	GZMK	0.141	***	0.013	0.688
**Resident memory T-cell**	GZMA	0.19	***	0.085	**
	IFNG	0.209	***	0.15	***
	CD69	0.2	***	0.084	**
	ITGAE	0.015	0.62	0.044	0.17
	CXCR6	0.252	***	0.159	***
**Exhausted T-cell**	MYADM	-0.067	*	-0.069	*
	HAVCR2	0.214	***	0.166	***
	TIGIT	0.248	***	0.159	***
	LAG3	0.206	***	0.163	***
	PDCD1	0.213	***	0.117	***
	CXCL13	0.229	***	0.16	***
**Resting Treg T-cell**	LAYN	0.056	0.0642	-0.041	0.199
	FOXP3	0.195	***	0.112	***
	IL2RA	0.288	***	0.224	***
**Effective Treg T-cell**	FOXP3	0.195	***	0.112	***
	CTLA4	0.247	***	0.172	***
	CCR8	0.182	***	0.139	***
	TNFRSF9	0.298	***	0.227	***
**Th1-like**	CXCL13	0.229	***	0.16	***
	HAVCR2	0.214	***	0.166	***
	IFNG	0.209	***	0.15	***
	CXCR3	0.167	***	0.055	0.0846
	BHLHE40	-0.161	***	-0.15	***
	CD4	0.22	***	0.127	***
	CCR7	0.2	***	0.093	**
**General memory T-cell**	SELL	0.192	***	0.086	**
	IL7R	0.261	***	0.162	***

### Prognostic potential of ceruloplasmin expression in BRCA patients stratified into subgroups according to the immune cell population

We examined whether ceruloplasmin influenced the survival of BRCA patients through its effects on immune infiltration. High ceruloplasmin expression in BRCA patient cohorts with decreased eosinophils and decreased mesenchymal stem cells was associated with worse RFS (Supplementary Figure 5D, 5F). By contrast, a marked correlation of high expression of ceruloplasmin and inferior RFS was found in the increased NK T cell cohorts (Supplementary Figure 5G). No obvious relationships were found between ceruloplasmin expression and RFS, with either increased or decreased CD4+ memory T cells, CD8+ T cells, B cells, macrophages, Treg cells, Th1 cells or Th2 cells in BRCA patients (Supplementary Figure 5A–5C, 5E, 5H–5J).

## DISCUSSION

In the current study, we performed bioinformatics analyses to comprehensively investigate the expression profiles, prognostic value, genetic mutations, and DNA methylation of ceruloplasmin and to analyze the correlations with immune infiltration in BRCA. We found that ceruloplasmin mRNA and protein expression was remarkably downregulated and correlated with sex, race, tumor clinical stage and pathological grade (Figures 1–4). We tried to determine the prognostic value of ceruloplasmin and its possible clinical translation for prediction of BRCA prognosis. Kaplan-Meier analysis demonstrated that ceruloplasmin could act as a sensitive and independent predictor of prognosis in BRCA patients (Figure 5). Moreover, increased DNA methylation in the ceruloplasmin gene was negatively correlated with ceruloplasmin expression and is therefore regarded as an unfavorable prognostic factor in BRCA patients (Figure 6).

As an essential micronutrient, iron plays crucial roles in cell survival and various physiological activities [23]. The mammalian dynamic equilibrium of iron is precisely regulated through complex processes including iron transport, storage and utilization in diverse cells and tissues [23]. Tumor cells usually require relatively high concentrations of iron to facilitate their increased DNA synthesis and rapid cell proliferation (relative to that in normal cells) [23, 24]. A growing body of research indicates that the expression levels of iron homeostasis-associated genes have been recognized as prognostic predictors and therapeutic targets for many cancers [24, 25]. The complex interactions among multiple iron-binding proteins, transporters, ferrioxidases and receptors enable the safe handling of iron *in vivo*. Some iron chelators have been developed as antitumor agents, such as deferasirox (DFX), Dp44mt, deferoxamine (DFO) and triapine, which can induce apoptosis in many types of cancer [26, 27]. Dp44mT exhibits anticancer activity in a number of cancers, such as leukemia, neuroblastoma, oral, lung, prostate and breast cancer, by promoting apoptosis, inducing cell cycle arrest and upregulating the expression of NDRG1 [27]. Similarly, DFX was also found to trigger apoptosis by suppressing the ER stress response and mTOR pathway [28, 29]. The potential benefit of iron-chelating agents was also observed in patients with leukemia and neuroblastoma in clinical studies [30]. Additionally, the combination of iron chelators with cyclophosphamide or cisplatin significantly increased the anticancer effects of these chemotherapeutics [27]. Cisplatin and radiotherapy with triapine, a synthetic iron chelator, improved the rate of metabolic complete response without significant toxicity in phase II clinical trials of uterine cervix or vaginal cancer [31].

Ceruloplasmin is a multicopper ferroxidase that mainly utilizes the redox activity of copper to oxidize ferrous iron, facilitating iron efflux via FPN1 [7, 8, 32]. Thus, the aberrant expression of ceruloplasmin has been reported in certain cancers. The serum levels of both ceruloplasmin and GPI-CP were remarkably increased in patients with lung cancer. CP can be heterotopically generated in lung adenocarcinoma (LUAD) cells [20]. The expression of ceruloplasmin is drastically upregulated in LUAD and significantly associated with clinicopathological stage [20]. High levels of ceruloplasmin are correlated not only with disease occurrence and invasiveness but also with worse outcomes in patients with lung cancer. Ceruloplasmin expression is also reported to be increased more than 10-fold in high-grade clear cell renal cell carcinoma samples [33, 34]. Ceruloplasmin expression cannot be detected in normal squamous epithelium and endocervical glands, butits expression is increased in cervical cancer subtypes, including squamous cell carcinoma and adenocarcinoma [21]. Furthermore, serum ceruloplasmin is utilized as a diagnostic biomarker for oral premalignancies and oral cancer [35]. In contrast, the expression of most iron-regulatory genes, including ceruloplasmin, is significantly lower in the tumor tissues of patients with hepatocellular carcinoma than in their adjacent normal liver tissues [22]. Four core fucosylated sites of ceruloplasmin were identified by mass spectrometry-based methodology [36]. The fucosylation of ceruloplasmin was significantly enhanced in alcohol-related hepatocellular carcinoma tissues compared with alcohol-related cirrhosis tissues [36]. Additionally, decreased ceruloplasmin and FPN1 expression was identified as an ACC-specific signature [21]. Additionally, consistent with our results, bioinformatics analyses revealed that ceruloplasmin and FPN1 may be involved in the immune response. However, the biological significance of ceruloplasmin dysregulation in cancer cells has not been completely verified and remains controversial. Here, we confirmed that both the mRNA and protein expression levels of ceruloplasmin were remarkably downregulated in BRCA through analysis of multiple databases. Decreased ceruloplasmin protein levels are also strongly related to several clinicopathological characteristics, such as subtype, age, race and tumor stage (Figure 4). These findings indicate the potential of ceruloplasmin as a sensitive biomarker for the diagnosis of BRCA.

In the present study, we identified many oncogenesis-associated pathways affected by ceruloplasmin, such as the HIF-1 signaling pathway, from the KEGG analysis (Figure 8). Ceruloplasmin is sensitive to oxygen and iron concentrations and is transcriptionally and HIF-dependently upregulated during hypoxia [37]. Ceruloplasmin is a direct target of SARI (basic leucine zipper ATF-like transcription factor 2) in dextran sodium sulfate (DSS)- and azoxymethane (AOM)-induced colon cancer. SARI promotes the proteasomal degradation of ceruloplasmin in the nucleus under normoxic and hypoxic conditions, thereby preventing the activation of the HIF-1α/VEGF axis. SARI knockout enhanced angiogenesis by regulating the expression of HIF-1α, VEGF and ceruloplasmin in mice [38]. In lung cancer cells, silencing ceruloplasmin enhanced the iron concentration and upregulated the activity of PHD1/2 to induce the hydroxylation of HIF-2 [39]. These results suggest that ceruloplasmin regulates HIF-2α activity through an iron/PHD cascade-dependent pathway. Additionally, miR-145-5p directly targets ceruloplasmin expression, and increased ceruloplasmin contributes to activation of the PHD/HIF-2α/VEGF-A axis to facilitate cancer growth and metastasis in LUAD [39]. In addition to HIF-related pathways, other signaling pathways have been reported to mediate the dysregulation of ceruloplasmin. Long noncoding RNA LINC00176 positively upregulates the expression of ceruloplasmin by recruiting transcription factor BCL3, a proto-oncogene in cancer. The LINC00176/BCL3/CP axis facilitates the epithelial-mesenchymal transition (EMT) process in ovarian cancer [40]. Cell-autonomous oncogenic driver paired box 8 (PAX8) is essential to maintain H3K27Ac at some genomic binding sites of metabolic genes [41]. PAX8 directly regulates ceruloplasmin expression by binding to a distal intragenic enhancer element. Additionally, ceruloplasmin is a predictive biomarker of PAX8 activity, and renal cell carcinoma (RCC) patients with high ceruloplasmin expression have a poor survival rate independent of genetic aberrations [41].

Recently, a growing number of studies have implied that cancer initiation and progression are not only regulated by genetic alterations but are also promoted by the TME [42]. Different types of immune cells in the TME are connected to metastasis, recurrence and prognosis. Recently, immune checkpoint inhibitors have successfully enhanced therapeutic efficacy in various types of cancer. Inhibitors of PD-1 and CTLA-4 have exhibited outstanding anticancer effects in multiple cancers, including BRCA [42, 43]. However, according to clinical observations, some patients remain insensitive to immunotherapy. Thus, new checkpoint immunosuppressants or combinations of checkpoint inhibitors with other targets may be critical to enhance the efficacy of immunotherapy. Exploring the interaction between tumors and immunity and identifying new immune-related therapeutic targets for BRCA treatment are critical issues. Previous studies have suggested that ceruloplasmin is an inflammatory response gene [15]. Consistent with these findings, our analyses indicated that multiple immune-related pathways are associated with ceruloplasmin (Figures 8, 9). In this study, we also assessed the effect of ceruloplasmin on the abundance of six major types of infiltrating immune cells and discerned distinct immune infiltration patterns in BRCA. Our analyses revealed that ceruloplasmin strongly affected the infiltration levels of major immune cells in BRCA, including some subtypes (Figure 10 and Supplementary Figure 3). T cells are very important for tumor surveillance. When we evaluated the relationships between ceruloplasmin expression and the gene markers of diverse T cells, we observed that ceruloplasmin was obviously associated with most markers of different subtypes of T cells (Table 3) [42, 43]. Interestingly, based on the correlation analyses, we demonstrated that ceruloplasmin is remarkably correlated with PD-1, PD-L1 and CTLA4 (Figure 10D and Supplementary Figure 4C). These data provide insights into the potential roles of ceruloplasmin in cancer immunology and immunotherapy.

Obesity is now a global public health problem, and its incidence is rapidly rising and related to the increased risk of BRCA [44]. The association between obesity and the risk of BRCA is complex and may vary according to race, subtype, menopausal status, and postmenopausal hormone therapy. However, a large amount of epidemiological evidence consistently implies that obesity is significantly linked with a higher risk of BRCA in postmenopausal women [44, 45]. Recent studies indicated that the expression level of ceruloplasmin, identified as an adipokine, is significantly increased in adipose tissues of obese individuals and obesity-associated cancer cells [46]. Obese patients exhibit characteristically higher ceruloplasmin serum levels [47]. Elevated ceruloplasmin levels are significantly associated with serum triglyceride and cholesterol levels in both men and women [48]. More importantly, improvement in obesity caused by energy restriction can significantly reduce the serum ceruloplasmin concentration in obese women, likely decreasing the risk of breast cancer in those subjects [49]. Steroid hormones, including estrogen and progestogen, could promote the proliferation and growth of hormone-sensitive BRCA cells by binding to their receptors (ER and PR) and inducing the expression of various specific genes. Hormone therapy, also known as endocrine therapy, can slow down or block the proliferation of hormone-sensitive BRCA cells by inhibiting the production of hormones or interfering with the action of hormones on cancer cells. Endocrine therapy has low toxicity and few side effects. If it is used properly, it can offer substantial benefits to elderly BRCA patients. A recent study showed a direct effect of estrogen on the production and/or release of ceruloplasmin in the liver using an animal model [50]. We also found that ceruloplasmin expression was obviously decreased in BRCA patients with hormone therapy (Supplementary Figure 6B). These results may explain some of the beneficial effects of hormone therapy. In the present study, our results clearly suggested that the expression of ceruloplasmin was dramatically reduced in ER+, PR+ and HER2+ subtypes compared to ER-, PR- and HER2- subtypes (Figure 3). Additionally, ceruloplasmin expression was markedly and positively associated with the expression of ERβ and EGFR, but negatively associated with the expression of ERα and ERBB2, indicating that there is a potential link between ceruloplasmin and hormone receptors (Supplementary Figure 6A). Additionally, there is a potential mechanistic connection among endocrine dysfunction, obesity, inflammation and breast cancer biology. The inflammatory cytokines/chemokines secreted by the adipose tissues in the local and/or system could activate the NF-kB, STAT3, HIF-1 and SIRT1 signaling pathways to promote invasion and metastasis of breast cancer [45]. Among these factors, SIRT1, an NAD^+^-dependent deacetylase, is involved in multiple biological processes, such as inflammation, mitochondrial biogenesis, glucose/cholesterol metabolism, cell senescence and consequent aging [51–53]. Recent studies demonstrate that SIRT1 is upregulated in breast cancer cells and tissues, facilitates cell proliferation, colony formation and cell migration *in vitro* and promotes tumorigenesis *in vivo* [54–56]. Many small molecule inhibitors of SIRT1 have been developed and exhibit encouraging anti-tumor effects against various cancers, including BRCA [57, 58]. Interestingly, we observed that ceruloplasmin was positively correlated with SIRT1 expression in BRCA (Supplementary Figure 6A), indicating that CP may affect the development of BRCA and the efficacy of immunotherapy through these immunity-related proteins and unknown signaling pathways.

Herein, we highlighted the potential prognostic relevance, genetic alterations and methylation status of ceruloplasmin in BRCA and identified ceruloplasmin as a modulator of tumor immune cell infiltration in BRCA patients. To our best knowledge, this is the first study in which BRCA patients with low ceruloplasmin expression are predisposed to a low immune infiltration status.

Although our findings could provide a useful indication of the involvement of ceruloplasmin in oncogenesis and TME in BRCA, there are several limitations should be noted. First, our results were only derived from the public open-access databases as a bioinformatics analysis. Although we confirmed the decreased expression of ceruloplasmin in BRCA cell lines and patient samples (Figure 4), the possible molecular mechanisms for ceruloplasmin in oncogenesis need further confirmation through experimentation and more patient datasets. Second, the relationships among ceruloplasmin, immune cell infiltration, and immune checkpoint genes were only evaluated by correlation analyses. Further verifications from both *in vitro* and *in vivo* experiments are required. Additionally, the relationships between ceruloplasmin and TMB, DNA methylation and other BRCA-related signaling pathways lack experimental data support and clarification of the mechanism. In summary, future studies are required to investigate the molecular mechanisms by which ceruloplasmin affects immune infiltration and to prospectively validate its clinical application.

## MATERIALS AND METHODS

### UALCAN

In the present study, the UALCAN online database (http://ualcan.path.uab.edu/analysis.html) was applied to explore the mRNA and protein levels of ceruloplasmin in BRCA tissues and normal tissues and its association with various clinical characteristics in BRCA.

### Oncomine

Ceruloplasmin mRNA expression in different cancers and the matched normal or adjacent tissues was compared according to the Oncomine database (https://www.oncomine.org/). In the present study, the parameters were set as follows: *P*-value <0.001, fold change > 2.

### Prognostic significance of ceruloplasmin expression in BRCA

The Kaplan–Meier Plotter database (https://kmplot.com/analysis/) was first used to explore the prognostic potential of ceruloplasmin in breast cancer. This database contains 6,234 breast datasets. The hazard ratios (HRs) with 95% confidence intervals (CI) and log rank *P*-values were obtained from the Kaplan–Meier Plotter database. Additionally, we used the PrognoScan (http://dna00.bio.kyutech.ac.jp/PrognoScan/index.html) database to further expand the prognosis-related investigation of ceruloplasmin expression. Overall survival (OS), recurrence/relapse-free survival (RFS), distant metastasis-free survival (DMFS) and postprogression survival (PPS) were employed using the PrognoScan database.

### GEPIA2

In this study, the GEPIA2 database (http://gepia2.cancer-pku.cn/#index) was utilized to investigate the expression pattern of ceruloplasmin in BRCA tissues and normal tissues. The “survival plots” module was used to examine the prognostic potential of ceruloplasmin, and “correlation analysis” was used to assess the association between ceruloplasmin and the expression of multiple immune gene markers.

### bc-GenExMiner (BC gene-expression miner) v4.5

The correlations between ceruloplasmin expression and multiple clinicopathological features of BRCA, including ER status, PR status, HER-2 status, SBR and NPI, were assessed through bcGenExMiner v4.5 (http://bcgenex.centregauducheau.fr/BC-GEM). The “prognostic module” was selected to calculate the prognostic and predictive values of ceruloplasmin expression in BRCA patients in the bcGenExMiner v4.5 database.

### cBioPortal

cBioPortal (https://www.cbioportal.org/) was used to examine the genetic alterations of ceruloplasmin in BRCA. The prognosis of BRCA patients with or without ceruloplasmin genetic alterations was also investigated using the cBioPortal database.

### SurvivalMeth

SurvivalMeth database (http://bio-bigdata.hrbmu.edu.cn/survivalmeth/) was applied to investigate the DNA methylation changes in the ceruloplasmin gene and the effect of DNA methylation on prognosis in BRCA patients.

### TIMER

The TIMER database was applied to estimate the relationship between ceruloplasmin and the infiltration levels of diverse immune cells in different BRCA subtypes. The abundance of six major tumor-infiltrating immune cells was investigated using the “Gene” module. The associations between ceruloplasmin expression and diverse immune cell marker sets were estimated through the ‘Correlation’ module.

### CIBERSORT algorithm

In the present study, the correlation between ceruloplasmin and the infiltration level of 22 types of tumor-infiltrating immune cells in BRCA was estimated using the CIBERSORT algorithm. A *p*-value less than 0.05 was used as the threshold to choose lymphocytes affected by ceruloplasmin.

### Human Protein Atlas (HPA) database

In our study, the immunohistochemistry (IHC) investigation of the expression of ceruloplasmin in BRCA was achieved through the HPA database (http://www.proteinatlas.org).

### GO, KEGG and GSEA

GO, KEGG and GSEA are powerful bioinformatics tools to assess the molecular function of ceruloplasmin in BRCA. GO depicts three biological concepts: BP, MF and CC. All these analyses were performed through the R package ClusterProfiler.

### Interaction network analysis

The gene-gene interactive network of ceruloplasmin was generated by the GeneMANIA database (http://www.genemania.org). The protein-protein interaction (PPI) network was generated using the STRING database (https://string-db.org/).

### Cell culture, real-time PCR and western blotting

Four cell lines, including the human normal breast epithelial cell line MCF-10A and three BRCA cell lines (MCF-7, MDA-MB-231 and MDA-MB-453), were cultured in DMEM (Gibco) consisted of 1% penicillin/streptomycin and 10% heat-inactivated FBS. Real-time PCR and western blotting were performed as previously described [59–61]. The primers for CP (forward, 5’-CCCTCAAACAAGTCTTACGCTCC-3’, reverse, 5’-CCAGGTAGAAGGTGGAATCCTC-3’) and S18 (forward, 5’-GCAGAATCCACGCCAGTACAAG-3’, reverse, 5’-GCTTGTTGTCCAGACCATTGGC-3’ are used in this study. The antibodies against ceruloplasmin (A7658, 1:1,000) and β-actin (66009-1-Ig, 1:1000) were purchased from ABclonal (Wuhan, China) and ProteinTech (Wuhan, China), respectively.

### Statistical analysis

The correlations of ceruloplasmin expression and TMB/MSI and the expression of other genes were evaluated by Spearman’s correlation. The multi-gene correlation map is generated using the R software package ggstatsplot, and visualized by the R software package ggplot2 (3.3.3). A p-value of <0.05 was considered statistically significant.

## Supplementary Material

Supplementary Figures

Supplementary Table 1
