# Supplementary material for: Ceruloplasmin correlates with immune infiltration and serves as a prognostic biomarker in breast cancer
Source: Aging (Albany NY). 2021 Aug 19;13(16):20438–67. doi: 10.18632/aging.203427 (PMC8436892; doi:10.18632/aging.203427)
Supplement: Supplementary Figures [file aging-13-203427-s001.pdf]

SUPPLEMENTARY FIGURES

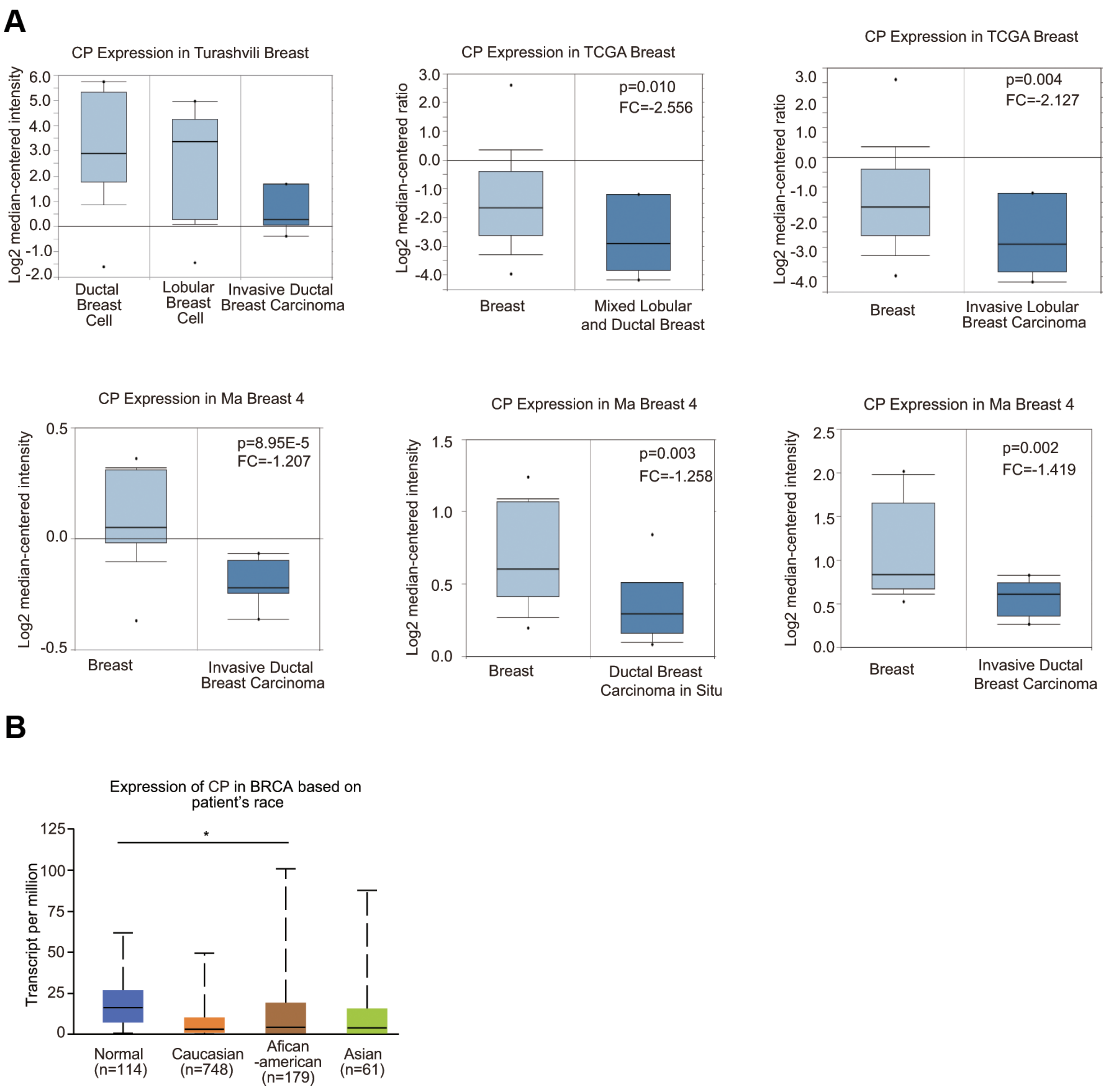

Supplementary Figure 1. (**A**) Box plots showing ceruloplasmin expression in the Turashvili breast, TCGA breast and Ma breast datasets. (**B**) Ceruloplasmin expression was estimated in BRCA patients according to race.

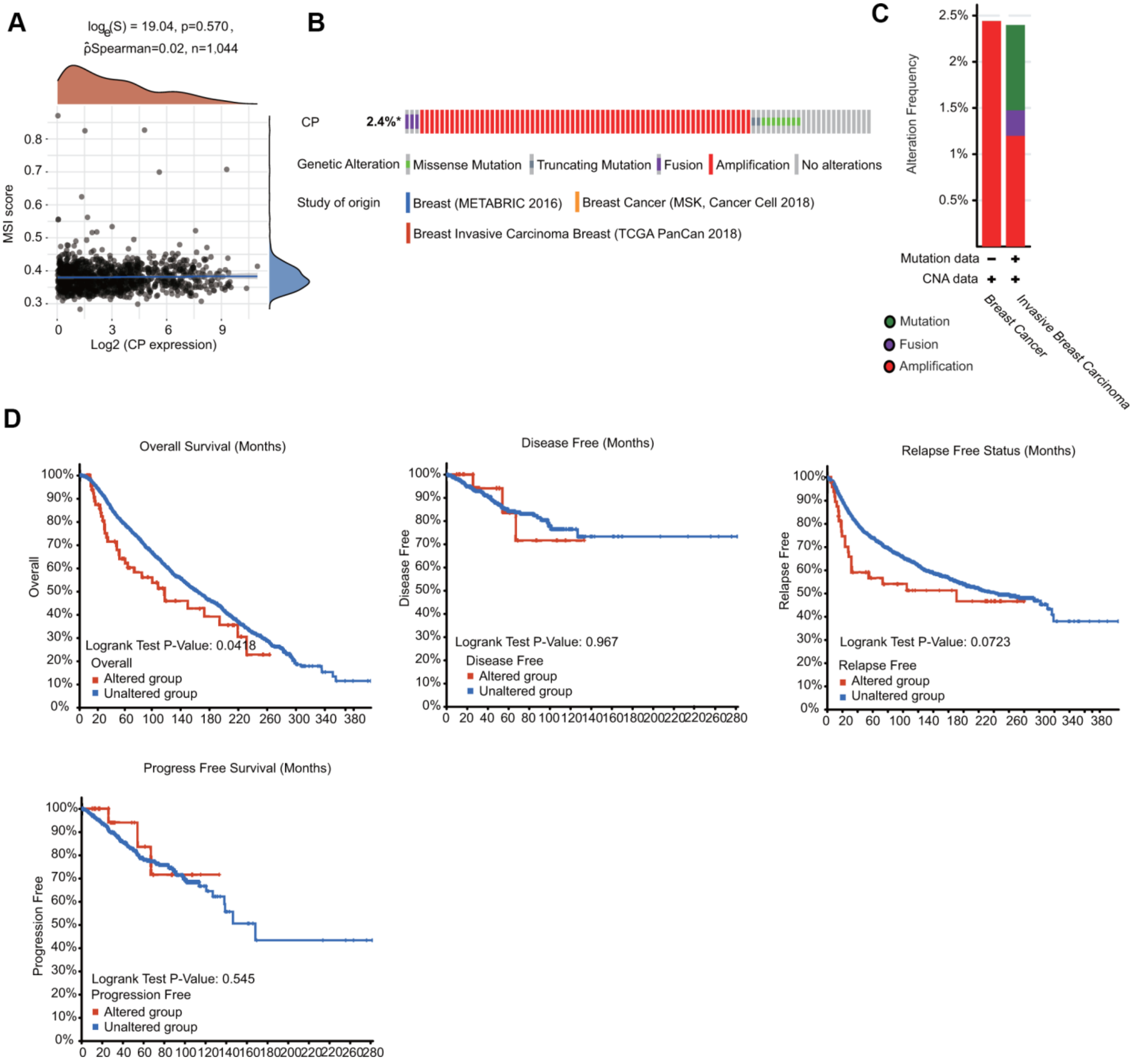

Supplementary Figure 2. Mutation frequency and survival analysis of ceruloplasmin in BRCA. (**A**) The correlation between ceruloplasmin expression and MSI in BRCA. (**B**) Visual summary of genetic alterations in the ceruloplasmin gene obtained from OncoPrint. (**C**) Summary of ceruloplasmin genetic alterations in HCC through cBioPortal. (**D**) Estimation of OS, RFS, DFS, and PFS in BRCA patients with or without ceruloplasmin genetic alterations.

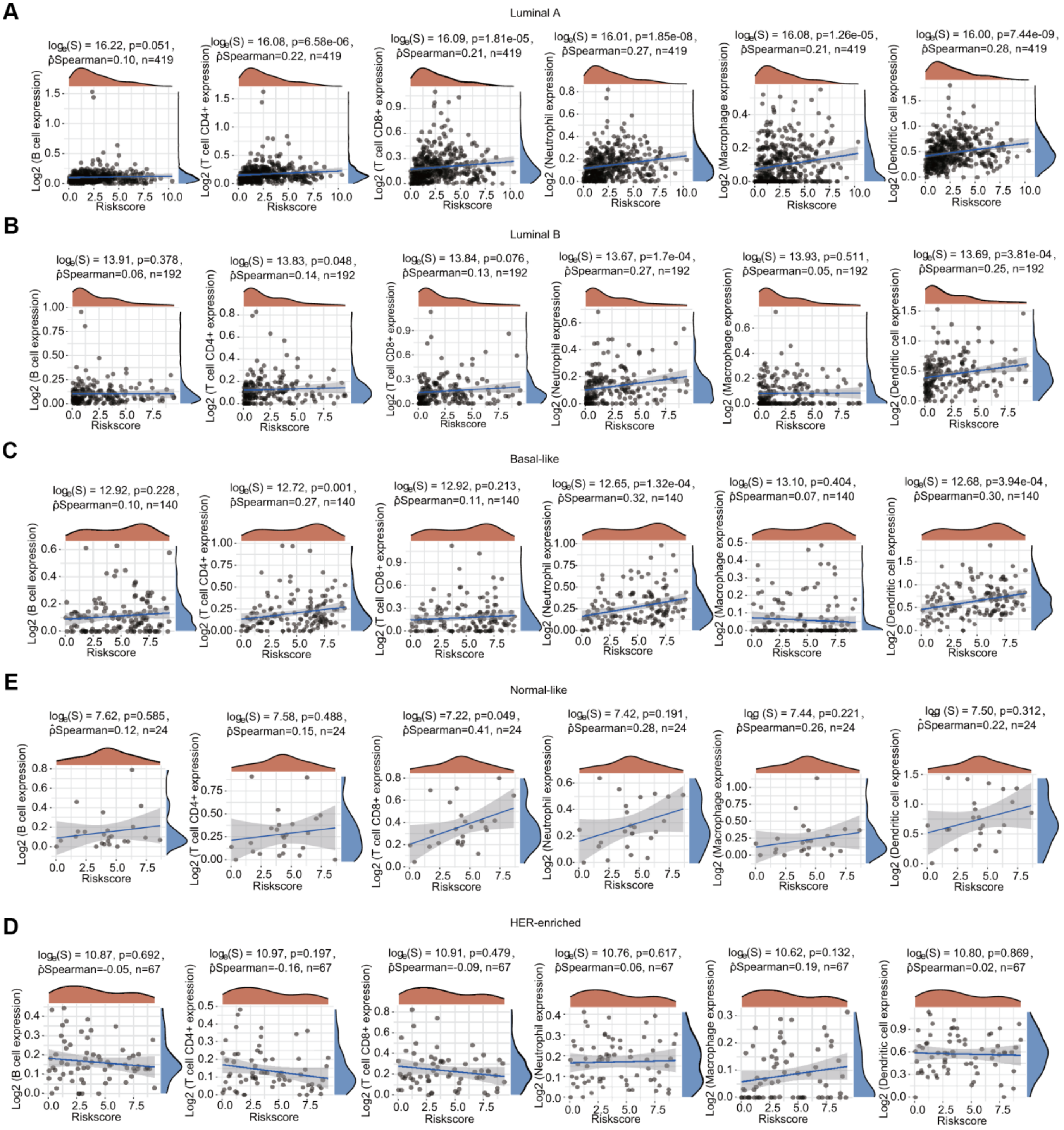

Supplementary Figure 3. Significant correlation of ceruloplasmin with the immune filtration level in different types of BRCA, including (**A**) luminal A, (**B**) luminal A, (**C**) HER2-enriched, (**D**) basal-like, and (**E**) normal-like.

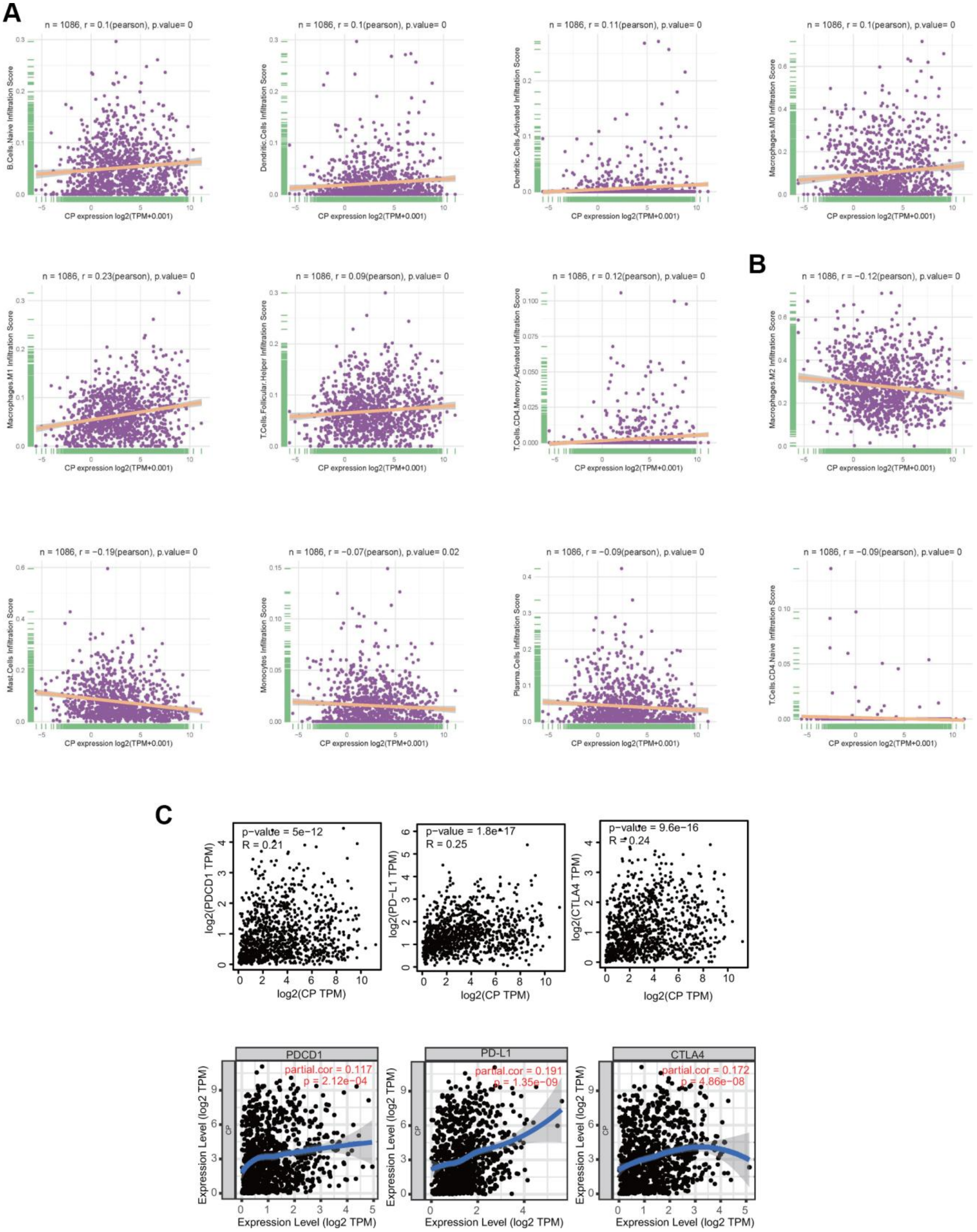

Supplementary Figure 4. The positive (**A**) and negative (**B**) correlations between ceruloplasmin expression and the infiltration of immune cells in BRCA using the CIBERSORT algorithm. (**C**) Correlations between ceruloplasmin and PD-1, PD-L1 and CTLA-4 expression in BRCA using the TIMER database.

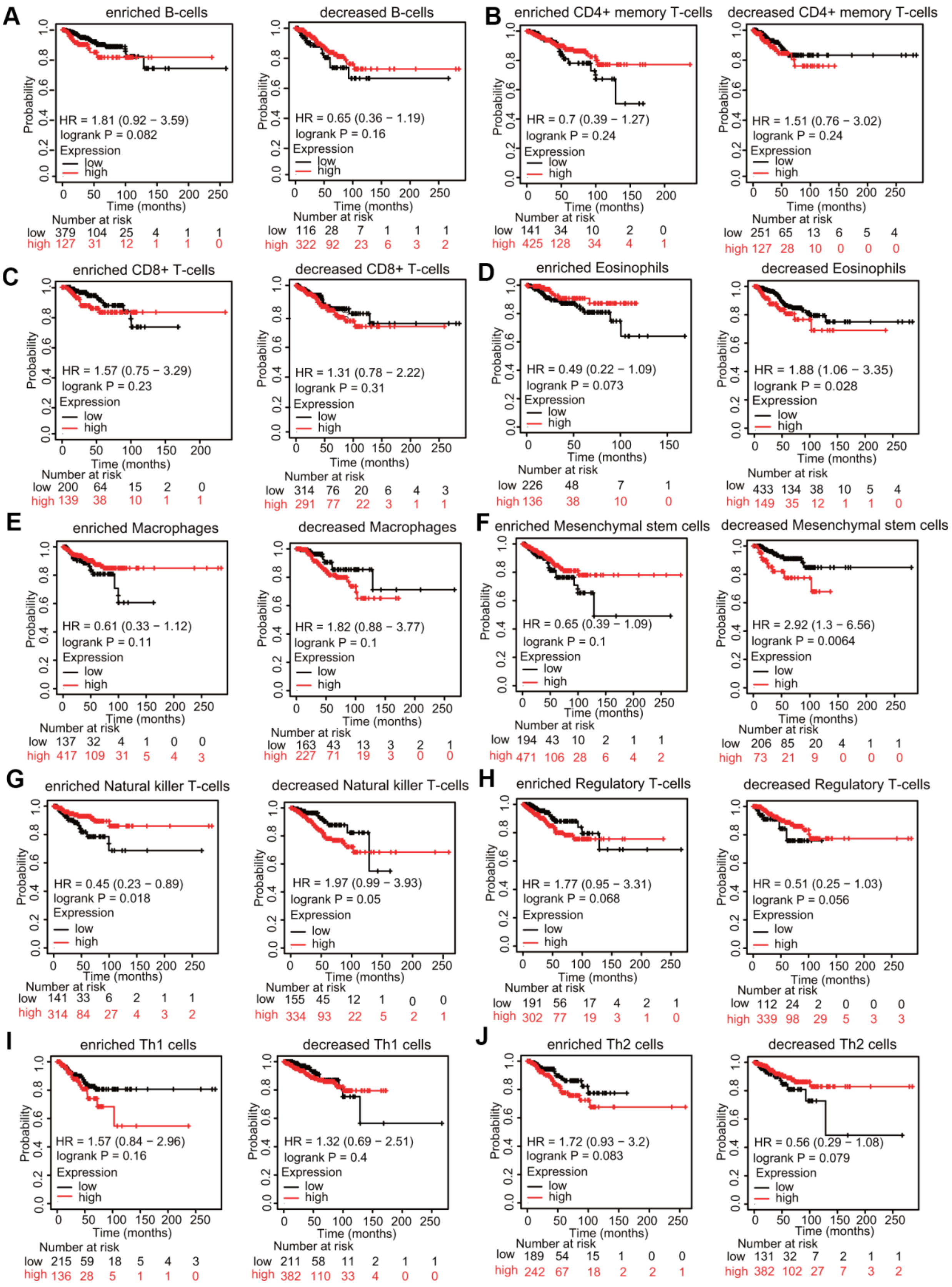

Supplementary Figure 5. (**A**–**J**) The relations between ceruloplasmin and RFS in diverse immune cell subgroups of BRCA patients were explored through Kaplan-Meier plotter.

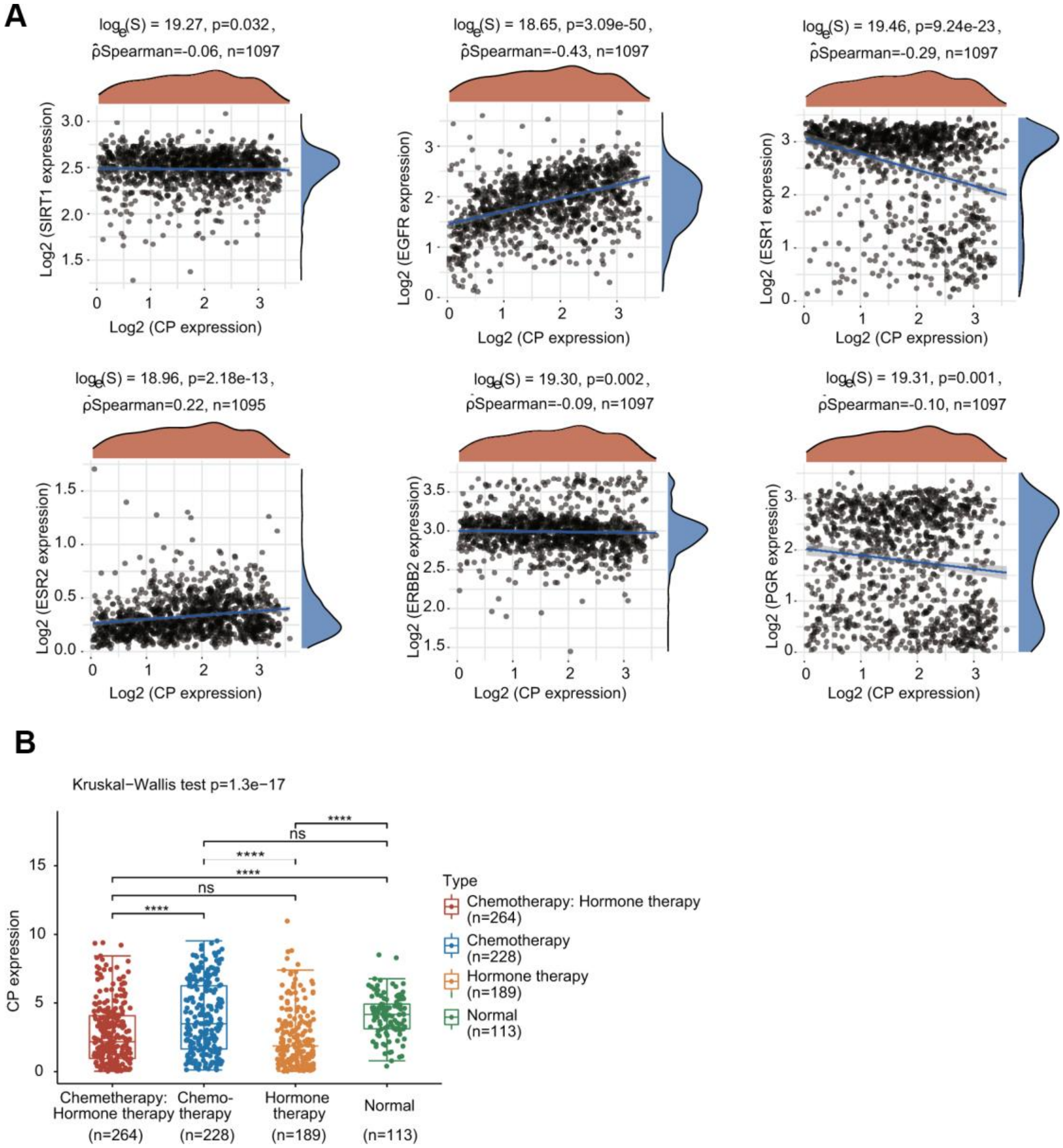

Supplementary Figure 6. (**A**) The correlation between the expression of ceruloplasmin and the expression of ER, PR, HER2 and SIRT1 was explored using the R software package ggstatsplot. (**B**) Ceruloplasmin expression in BRCA patients with different treatment was examined using the R software package ggplot2 and Kruskal-Wallis test.
